# An Air-Rechargeable Zn Battery Enabled by Organic–Inorganic Hybrid Cathode

**DOI:** 10.1007/s40820-023-01023-7

**Published:** 2023-02-16

**Authors:** Junjie Shi, Ke Mao, Qixiang Zhang, Zunyu Liu, Fei Long, Li Wen, Yixin Hou, Xinliang Li, Yanan Ma, Yang Yue, Luying Li, Chunyi Zhi, Yihua Gao

**Affiliations:** 1 Wuhan National Laboratory for Optoelectronics (WNLO) and School of Physics, Center for Nanoscale Characterization & Devices (CNCD), Huazhong University of Science and Technology (HUST), Wuhan, 430074 People’s Republic of China; 2 Guangxi Key Laboratory of Optical and Electronic Materials and Devices, College of Materials Science and Engineering, Guilin University of Technology, Guilin, 541004 People’s Republic of China; 3 Hong Kong Center for Cerebro-Cardiovascular Health Engineering, Hong Kong SAR, 999077 People’s Republic of China; 4 Hubei Key Laboratory of Critical Materials of New Energy Vehicles and School of Mathematics, Physics and Optoelectronic Engineering, Hubei University of Automotive Technology, Shiyan, 442002 People’s Republic of China; 5 Information Materials and Intelligent Sensing Laboratory of Anhui Province, Key Laboratory of Structure and Functional Regulation of Hybrid Materials of Ministry of Education, Institutes of Physical Science and Information Technology, Anhui University, Hefei, 230601 People’s Republic of China

**Keywords:** Air-rechargeable, MoS_2_/PANI Cathode, Desolvation shield, Energy storage mechanism, Zn batteries module

## Abstract

Self-charging power systems collecting energy harvesting technology and batteries are attracting extensive attention. To solve the disadvantages of the traditional integrated system, such as highly dependent on energy supply and complex structure, an air-rechargeable Zn battery based on MoS_2_/PANI cathode is reported. Benefited from the excellent conductivity desolvation shield of PANI, the MoS_2_/PANI cathode exhibits ultra-high capacity (304.98 mAh g^−1^ in N_2_ and 351.25 mAh g^−1^ in air). In particular, this battery has the ability to collect, convert and store energy simultaneously by an air-rechargeable process of the spontaneous redox reaction between the discharged cathode and O_2_ from air. The air-rechargeable Zn batteries display a high open-circuit voltage (1.15 V), an unforgettable discharge capacity (316.09 mAh g^−1^ and the air-rechargeable depth is 89.99%) and good air-recharging stability (291.22 mAh g^−1^ after 50 air recharging/galvanostatic current discharge cycle). Most importantly, both our quasi-solid zinc ion batteries and batteries modules have excellent performance and practicability. This work will provide a promising research direction for the material design and device assembly of the next-generation self-powered system.

**Supplementary Information:**

The online version contains supplementary material available at 10.1007/s40820-023-01023-7.

## Highlights

- The excellent performance of MoS_2_/PANI cathode is due to better conductivity and desolvation shielding.
- The self-charging zinc battery was successfully assembled that achieve deep self-discharge and long cycle life.
- The quasi solid state battery and battery module have excellent performance and practicability.

**Supplementary Information:**

The online version contains supplementary material available at 10.1007/s40820-023-01023-7.

## Graphical abstract

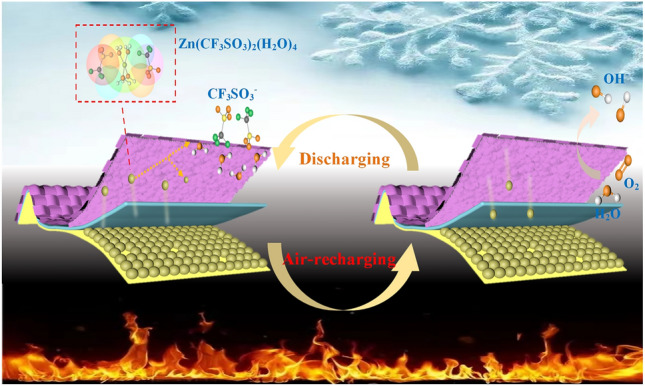

## Introduction

Up to now, many remote areas still do not have developed power grid or access to environmentally sound and affordable energy [1, 2]. Therefore, the development of self-charging power systems is very significant to solve the problem of energy poverty in underdeveloped areas. To solve this problem, the integrated systems have been developed, combining rechargeable battery (e.g., alkali batteries, zinc ion batteries (ZIBs)) with external energy collection systems (e.g., solar cells, thermobatteries, nanogenerators) [3–8]. However, under certain conditions, external energy collection systems are not always sustainable access, for example, solar cells need sunlight, thermobatteries need temperature difference and nanogenerators need additional mechanical energy [9–11]. In addition, the complex and large component of the integrated self-charging power systems is not suitable for the rapid development of portable and wearable electronics. Therefore, it is necessary to find a new type of self-charging power systems with simplified configuration and suitable for various environments.

Oxygen is known to be a rich source of energy from air and can be used to convert chemical energy into electrical energy through redox reactions. Therefore, combining the chemical energy of oxygen with the electrochemical energy of electrode material is an effective method to obtain self-charging batteries. Considering the instability of alkali batteries in the air [12–16], aqueous batteries seem to be the only option for self-charging batteries. In particular, ZIBs have recently stood out because the Zn anode possesses inherent merits such as high capacity (820 mAh g^–1^), low redox potential (− 0.76 V vs*.* SHE), high safety, environmental benignity and low cost [17–23].

At present, some researches have been made in the combination of ZIBs and oxygen to produce air-rechargeable ZIBs. For example, Chen et al. at first reported a air-rechargeable ZIBs based on GaVO cathodes, which can be charged by oxygen for 36 h to reach 1.05 V open-circuit voltage, providing a capacity of 239 mAh g^−1^ at 0.1 A g^−1^ [24]. However, the air-rechargeable ZIBs show limited cycle life (only five cycles), so the exploration of ZIBs with higher life was the next logical step. Subsequently, Wang et al. reported a air-rechargeable ZIBs based on poly(1,5-naphthaleneamine) cathode and alkaline electrolyte. Benefited from the weak coordination reaction between the cathode and cation, the cycle life of air-rechargeable ZIBs is improved (100 cycles). However, its discharge capacity is only 186 mAh g^−1^ at 0.2 A g^−1^ [25]. These works undoubtedly provide an effective strategy for the development of air-rechargeable ZIBs. However, it is still necessary to develop new air-rechargeable cathodes to obtain air-rechargeable ZIBs with both long life and high capacity.

As well known, among many ZIBs cathodes materials, organic–inorganic hybrid materials (OIHMs) have long cycle life and high capacity, benefiting from that organics can manage to enhance the electronic transfer, suppress the cathode dissolution and moderate the charge-shielding effect of Zn^2+^ [26–34]. Therefore, OIHMs are considered as the alternatives to cathodes, which evokes a new topic of whether these OIHMs can be used to fabricate air-rechargeable ZIBs. In particular, the most of reported OIHMs with higher reduction potential (> 0.9 V vs*.* Zn/Zn^2+^) such as V_2_O_5_/PANI [26, 27], C_2_H_8_N_2_V_7_O_16_ [28], MnO_2_/PANI [29], V_2_O_5_@PEDOT [30], PEDOT-NH_4_V_3_O_8_ [31], NVO-PEDOT [32], VO_2_/PPy [33] and VOPO_4_/PA [34] are not conducive to fast and reliable air recharging by oxygen. Therefore, up to now, the air-rechargeable performance of OIHMs with the presence of oxygen has never been reported.

Herein, we propose an air-rechargeable ZIB based on polyaniline-coated MoS_2_ cathodes (MoS_2_/PANI). The introduction of conductive polymer (PANI) coating of MoS_2_ not only improves the conductivity, but also induces the charge redistribution and structure changes at the interface to weaken the electrostatic interaction, thus promoting the diffusion of Zn^2+^. Accordingly, MoS_2_/PANI cathodes show the best electrochemical performance of zinc ion storage (304.98 mAh g^−1^ in N_2_ and 351.25 mAh g^−1^ in air at 0.50 A g^−1^) among the MoS_2_-based cathodes so far [25–42]. More importantly, the ex situ characterization of MoS_2_/PANI and MoS_2_ proved that PANI coated on MoS_2_ surface could play a desolvation role, which enables MoS_2_/PANI materials to have great capacity and minimal lattice strain in the discharging process. Benefited from the low reduction potential of the MoS_2_/PANI cathodes (0.71 V vs. Zn/Zn^2+^), the ZIB can be quickly and deeply air recharged by oxygen (316.09 mAh g^−1^ at 0.50 A g^−1^ after 24-h air recharging, i.e., 89.99% capacity retain of galvanostatic charge at 0.50 A g^−1^) and have long air recharging/galvanostatic discharging life (50 cycles). As a proof of concept, the quasi-solid zinc ion batteries (QSZIBs) assembled by MoS_2_/PANI cathode, zinc nanoflakes anode and polyacrylamide/polyethylene glycol/zinc trifluoromethanesulfonic acid (PAM/PEG/Zn(CF_3_SO_3_)_2_) hydrogel electrolyte demonstrates great electrochemical performance, excellent flexibility, high- and low-temperature stability and air-recharging ability. Finally, to further verify their practicality, a 3 × 3 battery module was successfully assembled and powered a pressure sensor and a smartphone. This work will provide a promising research direction for the material design and device assembly of the next-generation self-powered system.

## Results and Discussion

### Design Principle and Structural Characterizations

**Fig. 1:**
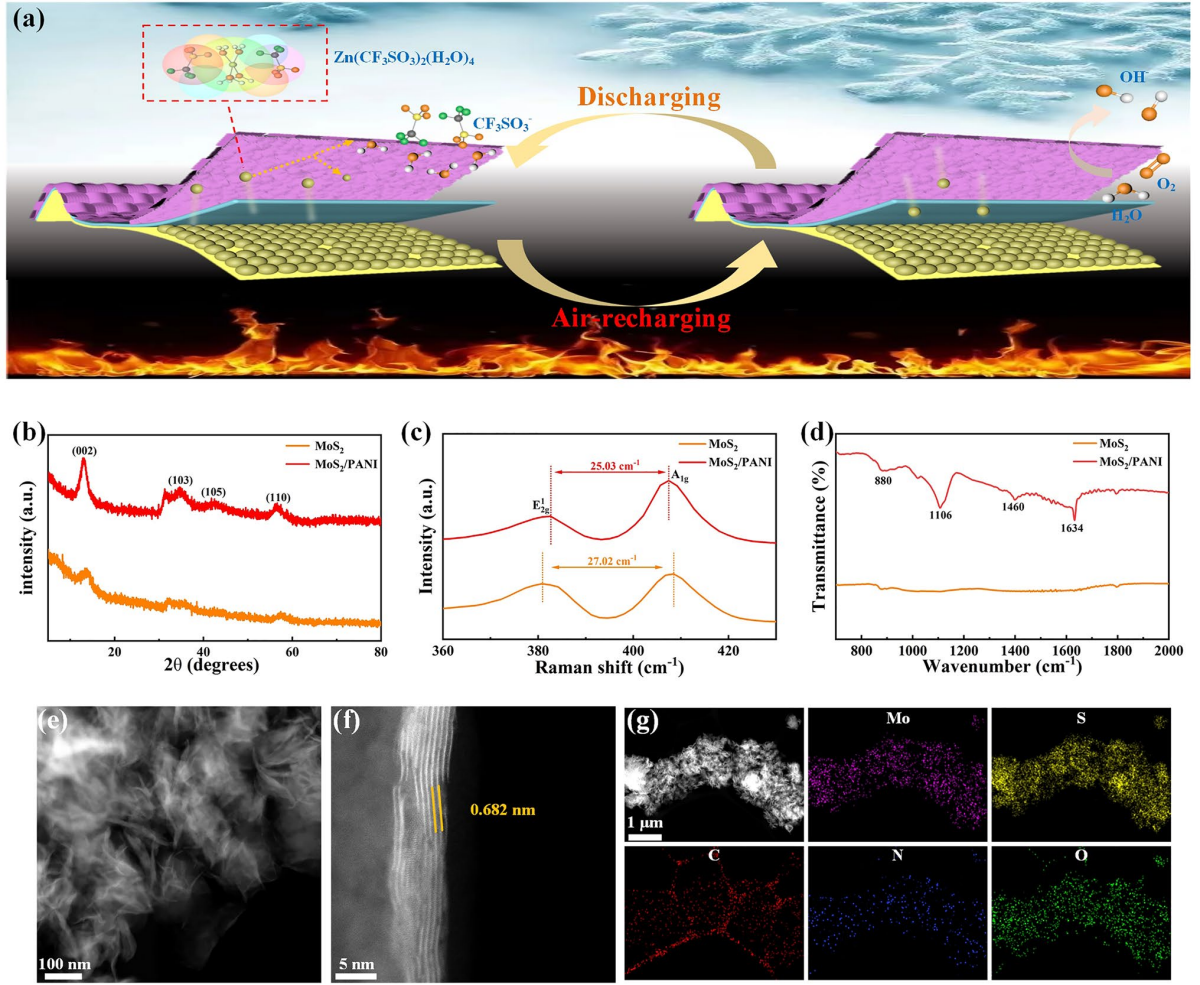
Structure of air-rechargeable QSZIBs and characterization of MoS_2_/PANI nanoflowers. **a** Structure of air-rechargeable QSZIBs, **b** XRD patterns, **c** Raman patterns and **d** FTIR spectrum of original MoS_2_ and MoS_2_/PANI. **e** Enlarged TEM image, **f** HTEM image and **g** STEM elemental mapping image of MoS_2_/PANI nanoflowers

The structure design, air recharging and galvanostatic discharging processes of the QSZIBs are shown in Fig. 1a. The QSZIBs consist of MoS_2_/PANI cathode, zinc nanoflakes anode and PAM/PEG/Zn(CF_3_SO_3_)_2_ hydrogel electrolyte. When discharged QSZIBs are exposed to air, Zn^2+^ was extracted from cathode in the presence of O_2_ and H_2_O. When QSZIBs is used to power the external circuit, the solvated Zn^2+^ moves from anode to cathode and is embedded into the cathode after being de-solvated on the cathode surface. The original MoS_2_ and MoS_2_/PANI nanoflowers were synthesized by simple hydrothermal method, and the successful synthesis was proved by a series of characterization. Figure 1b exhibits the X-ray diffraction (XRD) patterns of the original MoS_2_ (JCPDS No. 37–1492) and MoS_2_/PANI [43, 44]. It can be seen that compared with the original MoS_2_, the peak of MoS_2_/PANI shows a small angle shift, indicating that the most of PANI is coated on the surface of MoS_2_ and only a very extreme amount of PANI enters in interlayer. However, the diffraction peaks of PANI are not observed in the samples (Fig. S1a), probably because only a very small amount of PANI was introduced in the MoS_2_/PANI. The TG curves of MoS_2_ and MoS_2_/PANI in Fig. S2 indicate that the content of PANI in the MoS_2_/PANI is 5.27 wt%. The Raman spectra of original MoS_2_ and MoS_2_/PANI are shown in Fig. 1c. The interval between the *E*_2g_^1^ and *A*_1g_ peaks of MoS_2_/PANI decreased, indicating that the introduction of PANI weakened interaction between neighboring MoS_2_ layers [35]. The FTIR and XPS results further confirmed the successful synthesis of MoS_2_/PANI. As shown in Figs. 1d and S1b, the FTIR spectrum of MoS_2_/PANI nanoflowers also shows the peaks related to PANI, but with some offsets due to the presence of MoS_2_. As shown in Fig. S3, the full X-ray photoelectron spectrometer (XPS) spectrum of MoS_2_/PANI shows the signal of Mo, S, O, C and N which are attributed to MoS_2_ and PANI, respectively. As can be seen from the Mo 3*d* XPS spectra in Fig. S4a, the peak values of 228.9 and 232.1 eV with the spin energy separation of 3.2 eV were observed, which correspond to the Mo 3*d*_5/2_ and Mo 3*d*_3/2_ of Mo^4+^, respectively [45], while the obvious peak at 236.2 eV is associated with the Mo^6+^ due to MoS_2_ defects caused by the introduction of PANI. It is worth noting that there is also a weak Mo^6+^ peak in the original MoS_2_ (Fig. S5a), which may be related to the incomplete decomposition of raw materials [36]. The XPS spectrums spectra were fitted to analyze the phase composition of the original MoS_2_ and MoS_2_/PANI, which are both composed of 1 T-phase and 2H-phase (Figs. S4b, S5b and Table S1). It is worth noting that the strain of lattice mismatch caused by a small amount of PANI entering the interlayer of MoS_2_ is the reason that MoS_2_/PANI contains more 1 T phases than the original MoS_2._ The morphology and microstructure of original MoS_2_, PANI and MoS_2_/PANI were characterized by scanning electron microscopy (SEM). Compared with the original MoS_2_ and PANI (Fig. S6a, b), the MoS_2_/PANI exhibits smaller and fluffier nanoflowers (Fig. S6c). The microstructure of original MoS_2_, PANI and MoS_2_/PANI was further studied by transmission electron microscopy (TEM). The PANI consisting of C and N shows an amorphous phase (Fig. S7). As can be seen from the TEM images of MoS_2_/PANI nanoflowers in different magnifications in Figs. S8a and 1e, the MoS_2_/PANI nanoflowers are assembled from nanosheets around 100 nm, which display much thinner and smaller nanoflowers than the original MoS_2_ (Fig. S9a, b). As shown in Figs. 1f and S9c, the high-resolution TEM (HRTEM) images show that the layer spacing of MoS_2_ (002) in MoS_2_/PANI increased from 0.620 to 0.682 nm, indicating that an extreme small amount of PANI entered in MoS_2_ layers, which is similar to the XRD results (Fig. 1b). Meanwhile, the HAADF-TEM images of original MoS_2_ and MoS_2_/PANI visually prove that the phase species are both composed of 1 T-phase and 2H-phase (Figs. S8d and S9d). Typical interfaces of MoS_2_ and PANI are also observed in Fig. S8b-d, which provides compelling evidence for the coating of this PANI. The STEM elemental mapping image in Fig. 1g reveals the homogeneous distribution of Mo, S, C, N and O elements along the MoS_2_/PANI nanoflowers.

### Electrochemical Performances and Electrode Process Kinetic

**Fig. 2:**
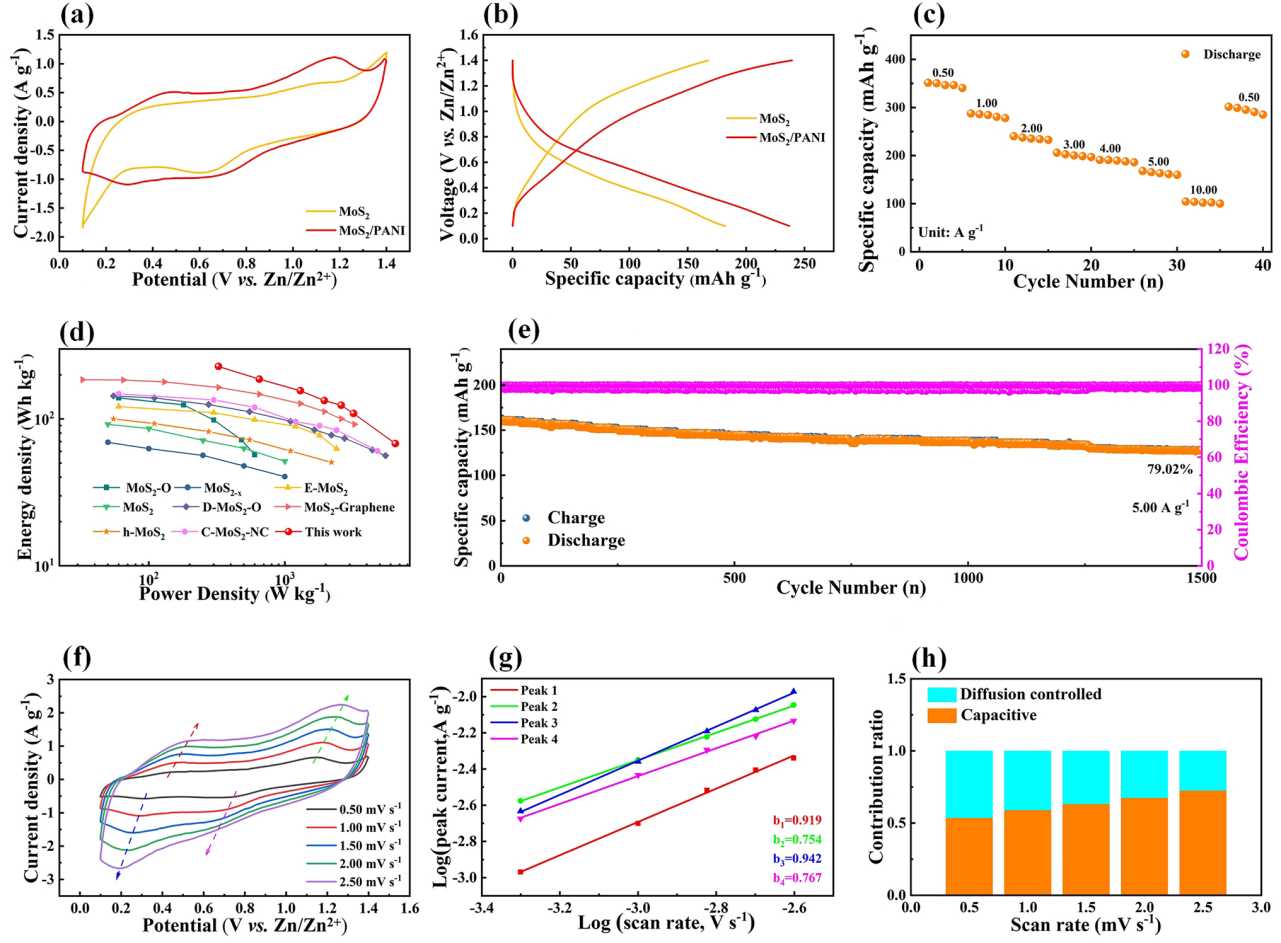
Electrochemical performances and electrode process kinetic of MoS_2_/PANI cathodes in air. **a** CV curves at a scan rate of 1.0 mV s^−1^ and **b** GCD curves at a current density of 2.0 A g^−1^ of the MoS_2_/PANI cathodes and MoS_2_ cathodes. **c** Rate capability, **d** energy and power density plot, **e** cyclic life and Coulomb efficiency and **f** CV curves at different scan rates from 0.50 to 2.50 mV s^−1^ of the MoS_2_/PANI cathodes. **g** Log (i) versus log (v) plots and **h** contribution ratios of diffusion-controlled and capacitive capacities at different scan rates of the MoS_2_/PANI cathodes

The electrochemical performance of MoS_2_/PANI was evaluated by a standard two-electrode system in air. The CV curves at 1.0 mV s^−1^ and GCD curves at 2.0 A g^−1^ of original MoS_2_, PANI and MoS_2_/PANI are shown in Figs. 2a, b and S10a, b. The MoS_2_/PANI cathodes show the maximum CV area and specific capacity, indicating the excellent modification effect of PANI on MoS_2_. The Nyquist plot, equivalent circuit and corresponding resistances of the original MoS_2_, PANI and MoS_2_/PANI are shown in Fig. S11a, b and Table S2, respectively, indicating that the introduction of PANI can effectively improve the electrochemical reaction kinetics of MoS_2_. As shown in Fig. S12, we further studied the MoS_2_/PANI cathodes with different amounts of PANI and found that both too high and too low amount of PANI lead to the deterioration of MoS_2_/PANI performance, which may be due to the fact that more or less PANI is not conducive to the formation of MoS_2_/PANI nanoflowers (Figs. S13 and S14). The specific capacity of the above samples was further tested at current densities from 0.50 to 10.00 A g^−1^ (Figs. S15-S18 and 2c) to prove that MoS_2_/PANI cathodes had the best electrochemical performance. In addition, Fig. 2c also shows the excellent rate capability of MoS_2_/PANI cathode (the specific capacity at 0.50, 1.00, 2.00, 3.00, 4.00, 5.00 and 10.00 A g^−1^ is 351.25, 287.56, 239.93, 205.80, 190.84, 167.78 and 104.44 mAh g^−1^, respectively). As notably highlighted in Fig. 2d, the MoS_2_/PANI cathodes manifest higher energy densities over a wider power density range compared to the recently reported MoS_2_-based cathodes for AZIBs, which has the power density and energy density as high as 6500.0 W kg^−1^ and 228.3 Wh kg^−1^, respectively. To illustrate their excellent stability, the MoS_2_/PANI cathodes were tested at a current density of 5.00 A g^−1^. After 1500 charge/discharge cycles, the capacity retention rate of the MoS_2_/PANI cathodes was 79.02% (Fig. 2e). The morphology and structure of MoS_2_/PANI cathodes before and after the cycle were characterized by SEM to prove the stability of MoS_2_/PANI cathodes. Figure S19b shows SEM images of MoS_2_/PANI cathodes after 1500 GCD cycles (charged to 1.40 V), which are basically unchanged from original morphology and structure (Fig. S19a), indicating that the ion insertion/extraction behavior during the cycle is reversible.

Then the energy storage mechanism of MoS_2_ and MoS_2_/PANI was studied. As shown in Figs. 2f and S20a, the electrochemical reaction kinetics of MoS_2_ and MoS_2_/PANI cathodes were revealed by CV curves at different sweep rates from 0.50 to 2.50 mV s^−1^. The electrochemical reaction kinetics can be assessed by Eq. 1 [46, 47]:

1 $$ i \, = \, av^{b} $$

As shown in Figs. 2g and S20b, the *b* values reveal that the electrochemical reaction kinetics of both MoS_2_ and MoS_2_/PANI cathodes are composed of capacitive and diffusion controlled. At a specified scan rate, the capacitive contribution and the diffusion-controlled contribution can be further evaluated according to Eq. 2 [48, 49]:

2 $$ i\left( v \right) \, = \, k_{1} v \, + \, k_{2} v^{1/2} $$

As shown in Figs. 2h and S20c, with the increase in scan rate, the ratio of the capacitive contribution process increases gradually, and the capacitive process plays a leading role in the total process. It is worth noting that the diffusion-controlled contribution of MoS_2_/PANI is always greater than that of MoS_2_ at all scan rates, indicating that the high capacity of MoS_2_/PANI is mainly due to ion diffusion rather than ion adsorption (Fig. S21).

### Galvanostatic Intermittent Titration Technique and Density Functional Theory

**Fig. 3:**
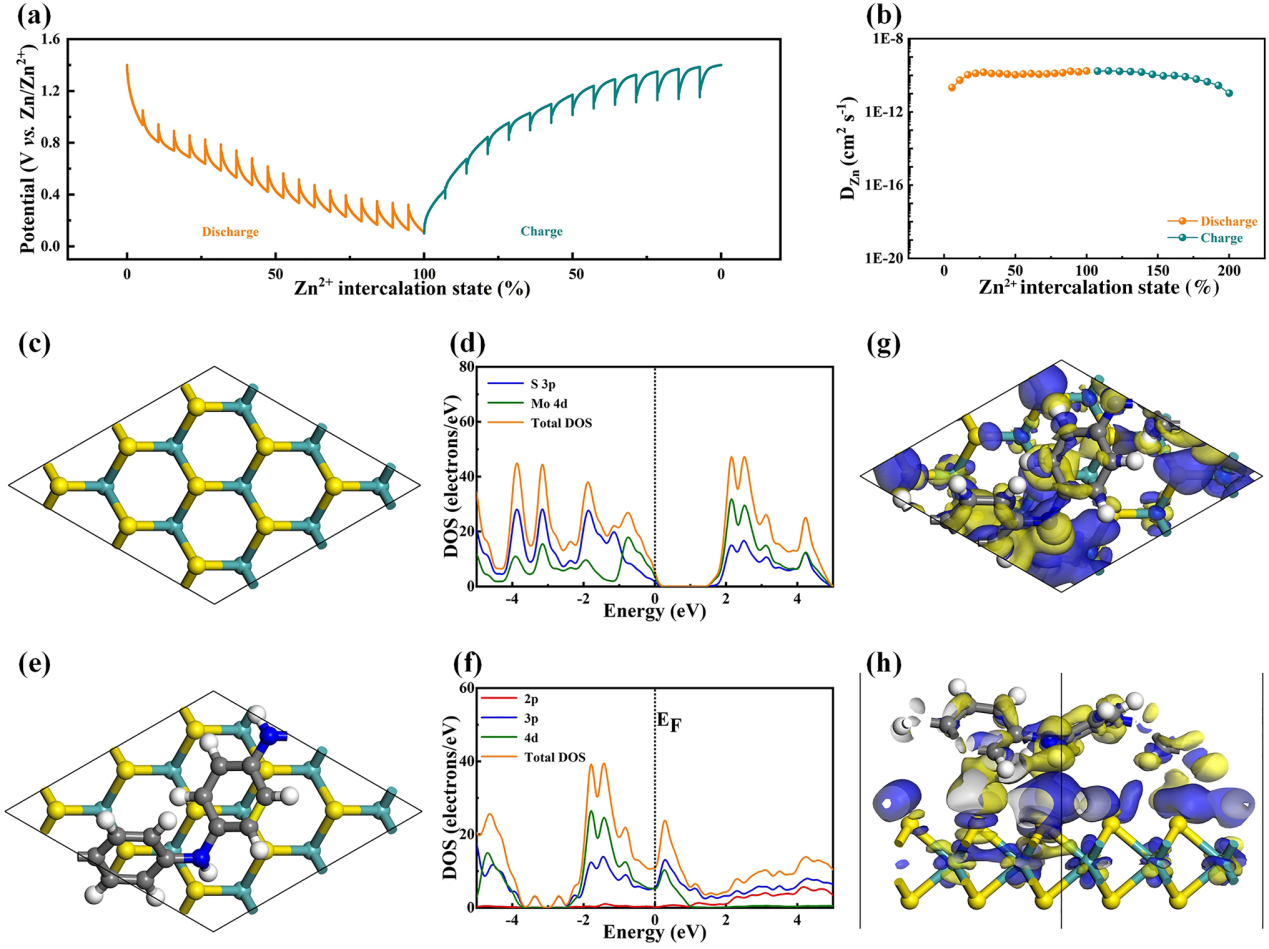
GITT and DFT of MoS_2_/PANI cathodes. **a** GITT and **b** D_zn_ of MoS_2_/PANI cathodes. **c** The initial model establishment of MoS_2_ and **d** corresponding density map. **e** The model establishment of MoS_2_/PANI and **f** corresponding density map. **g, h** The differential charge diagram of MoS_2_/PANI

The reaction kinetics of MoS_2_/PANI cathodes can be further studied by GITT (Fig. 3a) to estimate the Zn^2+^ diffusion coefficient (*D*_Zn_). As shown in Fig. 3b, the *D*_Zn_ calculated by galvanostatic intermittent titration technique (GITT, calculation details are described in Experimental Section) in the charge and discharge process consistently reveals a *D*_Zn_ values range of 10^−11^–10^−9^ cm^2^ s^−1^. The *D*_Zn_ values achieved in this work are higher than MoS_2_ and previously reported manganese and vanadium-based oxide cathodes for AZIBs (Fig. S22 and Table S3). The reaction kinetics of MoS_2_ and MoS_2_/PANI were also studied by density functional theory (DFT) calculation. The disadvantage of MoS_2_ as cathode material of ZIBs is its weak reaction kinetics due to its poor conductivity. The initial model establishment of MoS_2_ and corresponding density map are shown in Fig. 3c, d, respectively. It can be seen that the original MoS_2_ has a relatively large band gap, which is similar to the previous report [50–52]. Therefore, the transfer of electrons from valence to conduction band requiring a lot of energy indicates its poor conductivity, which limits its performance. The model establishment of MoS_2_/PANI and corresponding density map are shown in Fig. 3e, f, respectively. It can be seen that the state density near the Fermi level of MoS_2_/PANI increases, electrons are easier to transition from the valence band to the conduction band, and the introduction of PANI enhances the conductivity of MoS_2_/PANI. As shown in Fig. 3g, h of differential charge diagram of MoS_2_/PANI, the PANI and MoS_2_ base have obvious charge transfer, which further indicates that PANI can have relatively strong interaction with MoS_2_ after insertion. Therefore, after the incorporation of PANI, PANI will combine with the substrate material MoS_2_ and further improve the conductivity of the material. In addition, DFT calculation also proves that the increase of the layer spacing reduces the zinc ion transport barrier from 0.99 to 0.39 eV (Fig. S23).

### Energy Storage Mechanism

**Fig. 4:**
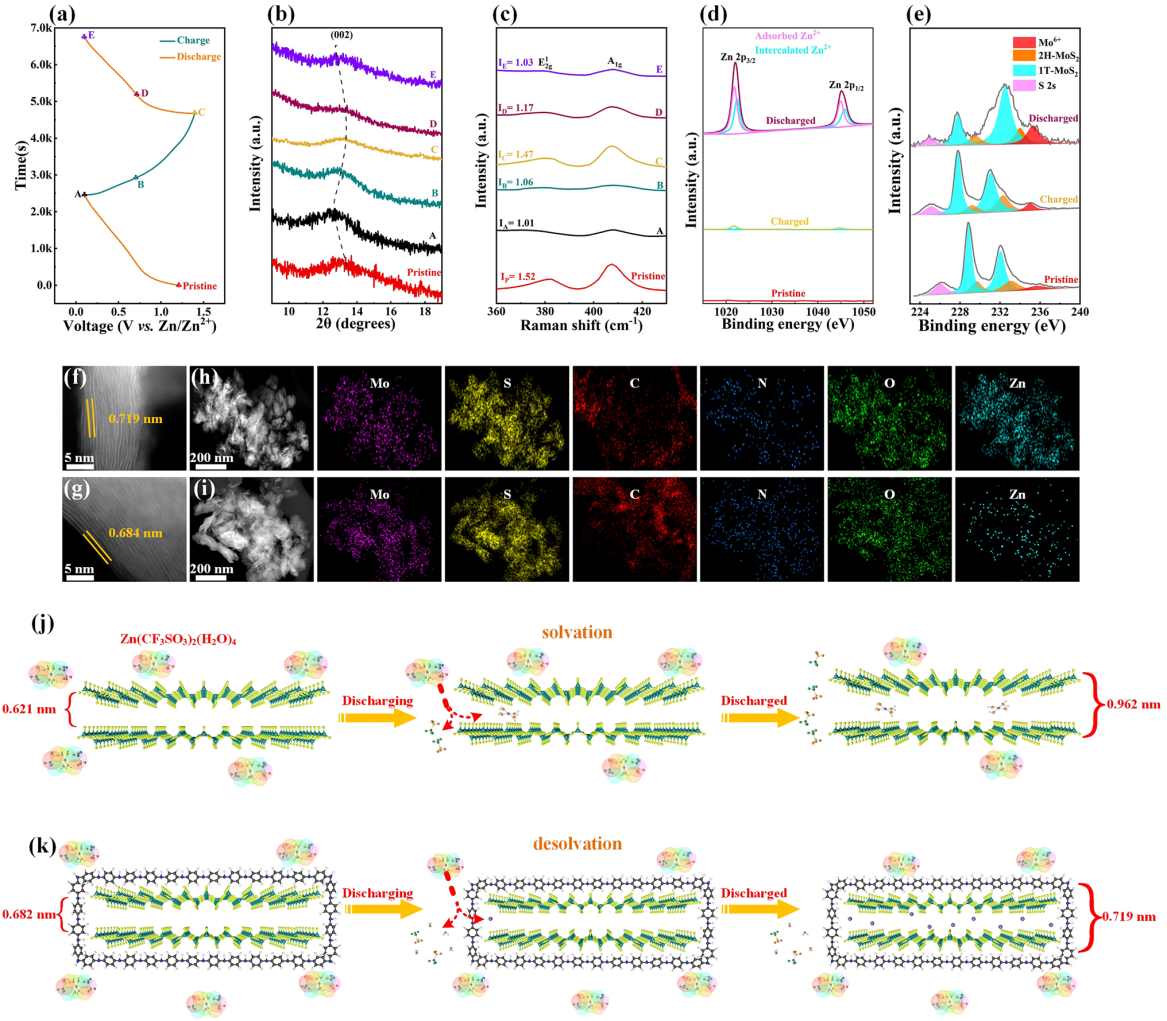
Zn storage mechanism of MoS_2_/PANI cathodes. **a** Initial discharge/charge curve at 0.50 A g^−1^, the marked states are selected for ex situ tests. *Ex situ* **b** XRD patterns, **c** Raman patterns, XPS spectra of **d** Zn and **e** Mo. **f, g** HRTEM and **h, i** STEM element mapping images of the fully discharged and charged MoS_2_/PANI cathodes. **j, k** The solvation and desolvation of MoS_2_ and MoS_2_/PANI

In order to clarify the energy storage mechanism, MoS_2_/ PANI cathode was tested in N_2_ to exclude external interference factors (such as air recharging). As shown in Figs. S24 and S25, the MoS_2_/PANI cathodes exhibit better cycling stability and lower capacity in N_2_. However, for layered materials, the interlayer spacing determines the storage capacity of zinc ions (Fig. S26), so MoS_2_/PANI cathodes may have some special energy storage mechanisms. In order to clarify the energy storage mechanism of MoS_2_/PANI cathode, the structural evolution of the MoS_2_/PANI cathode was characterized by ex situ XRD. The marked states (points A–E) during charge/discharge were selected for ex situ XRD tests (Fig. 4a). When the MoS_2_/PANI cathode discharged, the peak of (002) gradually shifts toward a smaller angle (Fig. 4b), reflecting the interlayer expansion caused by Zn^2+^. During charging (from state A to state C), the peak of (002) gradually recovers to the initial position, indicating that the ions insertion/extraction of MoS_2_/PANI cathode was highly reversible. It is worth noting that during the discharge process the peak of (002) moves very a little toward the lower angle, indicating that the interlayer expansion of MoS_2_/PANI cathode during charging and discharging process is very small. Generally speaking, when materials are discharged, they exhibit small interlayer expansion mainly for the following reasons: (1) limited zinc ion embedding; (2) other small ions interlayer contribution capacity; (3) there is desolvation phenomenon. First, the Inductive Coupled Plasma Emission Spectrometer (ICP) analyses of MoS_2_/PANI cathode at fully discharged and charged were tested. As shown in Table S4, the ratio of Zn to Mo atoms before and after MoS_2_/PANI charging and discharging is 0.8474 and 0.0237, which proves that the capacity of MoS_2_/PANI is mainly contributed by zinc ion intercalation. Then, we verify that the H^+^ insertion has a little contribution to the cathode capacity (Fig. S27). However, the presence of Zn^2+^ in 2.0 M Zn(CF_3_SO_3_)_2_ takes the form of larger Zn(CF_3_SO_3_)_2_(H_2_O)_4_ (inserted form is Zn(H_2_O)_4_^2+^), which usually causes larger interlayer expansion when inserted in electrode materials with smaller interlayer spacing [53–56]. Therefore, we supposed that when Zn(H_2_O)_4_^2+^ is inserted in MoS_2_/PANI cathode, there is a mechanism of desolvation on the surface of electrode material, which causes Zn(CF_3_SO_3_)_2_(H_2_O)_4_ to change into smaller Zn^2+^. Therefore, further tests were carried out to prove above conjecture and clarify the storage mechanism of MoS_2_/PANI cathodes. The *ex situ* Raman spectra of MoS_2_/PANI cathode at different charge and discharge states were collected (Fig. 4c). When MoS_2_/PANI cathode was discharged to 0.10 V, the intensity ratio of *A*_1g_/*E*^1^_2g_ decreased (*A* and* E*), indicating that ions had been inserted into the interlayer of MoS_2_/PANI cathode. When MoS_2_/PANI cathode was charged to 1.40 V, the intensity ratio of *A*_1g_/*E*^1^_2g_ recovered to 1.47 due to the extraction of ions. The intensity ratio of *A*_1g_/*E*^1^_2g_ changes slightly during charging and discharging process of MoS_2_/PANI cathode, which are consistent with the results of *ex situ* XRD. In order to prove that Zn^2+^ rather than other ions inserted MoS_2_/PANI cathode, XPS spectrum of the original, fully discharged and charged states has been studied. As shown in Fig. 4d, no Zn signal was detected in the Zn 2*p* XPS spectrum of the original electrode. When MoS_2_/PANI cathode was fully discharged, two pairs of Zn 2*p* signals can be observed, corresponding to the Zn^2+^ absorbed on the electrode surface and the Zn^2+^ inserted in electrode, respectively. After fully charging to 1.40 V, a pair of weak Zn signals were still observed due to the adsorption of residual Zn(CF_3_SO_3_)_2_. Spectra and fitting results of XPS in different states in Mo 3*d* of MoS_2_/PANI cathode are shown in Fig. 4e and Table S1. The Mo 3*d* XPS deconvolution shows that the pristine electrode contains 65.82% 1 T-MoS_2_ and 29.18% 2H-MoS_2_, along with 5.00% Mo^6+^ caused by surface oxidation. After being discharged to 0.10 V, the proportion of 2H-MoS_2_ is reduced to 18.78% (against 65.74% for 1 T-MoS_2_ and 15.48% for Mo^6+^), illustrating the phase transition from 2H- to 1 T-MoS_2_ induced by Zn^2+^ insertion. It is worth noticing that the 1 T-MoS_2_ could be easily oxidized (during the XPS testing process), especially after electrochemical activation [29], which explains the dramatically strengthened Mo^6+^ signal and the reduced 1 T-phase content. After charging to 1.40 V, the original content of 2H-MoS_2_ is finely restored, indicating the highly reversible phase transition triggered by Zn^2+^ insertion/extraction. The reversible phase transition between 1 T-MoS_2_ and 2H-MoS_2_ during Zn^2+^ insertion/extraction was confirmed. The *ex situ* HRTEM image (Fig. 4f, g) visually shows the fringe distance of 0.719/0.684 nm at the insertion/extraction of Zn^2+^, which is in good agreement with the XRD results. STEM elemental mapping images reveal the uniform distribution of Mo, S, C, N, O and Zn elements in the fully discharged electrode (Fig. 4h) and fully charged electrode (Fig. 4i), indicating the reversible insertion/extraction of Zn^2+^. Based on the above discussion, the storage mechanism of MoS_2_/PANI cathode is summarized as the reversible insertion/extraction of Zn^2+^ formed by the desolvation of Zn(CF_3_SO_3_)_2_(H_2_O)_4_ on the electrode surface rather than Zn(H_2_O)_4_^2+^. To investigate whether PANI plays an important role in the desolvation of Zn(CF_3_SO_3_)_2_(H_2_O)_4_, the Zn storage mechanism of the original MoS_2_ electrode has also been studied (Figs. S28 and S29). It is found that the peak of (002) has obvious shift to lower angle and the layer spacing increases to 0.962 nm. In addition, it is noteworthy that no significant change in the peak intensity of F 1* s* XPS spectrum was detected in the full charging and discharging of original MoS_2_ and MoS_2_/PANI cathode, indicating that CF_3_SO_3_^2−^ really did not inserted in the electrode and the detected F 1* s* spectra were attributed to the adsorption of electrolyte (Fig. S30). However, when the original MoS_2_ cathode was fully charged and discharged, the detected O 1* s* XPS spectral peak intensity changed significantly, indicating that Zn(H_2_O)_4_^2+^ may be inserted into the electrode (Fig. S31). Energy storage mechanism of original MoS_2_ and MoS_2_/PANI cathode in discharge process is shown in Fig. 4j, k. For the original MoS_2_, Zn(H_2_O)_4_^2+^ formed by the decomposition of Zn(CF_3_SO_3_)_2_(H_2_O)_4_ on the surface of MoS_2_ was directly inserted into MoS_2_, resulting the layer spacing increases. For MoS_2_/PANI cathode, Zn(CF_3_SO_3_)_2_(H_2_O)_4_ will first form coordination with PANI on the surface. Benefits from the synergistic effect of Zn^2+^ and π electrostatic interaction, the solvent shell around the Zn^2+^ will collapse in the transmission process [57, 58]. Finally, Zn^2+^ are separately extracted into the cathode material through PANI (Fig. S32). Therefore, MoS_2_/PANI cathode has a very small layer spacing increases after discharge and provides a very large capacity. Based on the above results and discussion, the overall electrochemical reaction of the ZIBs based on MoS_2_/PANI cathode can be described as follows:

Cathode:

3 $$ {\text{MoS}}_{{2}} /{\text{PANI }} + \, 0.{\text{85 Zn}}^{{{2} + }} + { 1}.{\text{7e}}^{ - } \leftrightarrow {\text{ MoZn}}_{{0.{85}}} {\text{S}}_{{2}} /{\text{PANI}} $$

Anode:

4 $$ 0.{\text{85 Zn}} \leftrightarrow 0.{\text{85Zn}}^{{{2} + }} + {1}.{\text{7e}}^{ - } $$

Overall:

5 $$ {\text{MoS}}_{{2}} /{\text{PANI }} + 0.{\text{85 Zn }} \leftrightarrow {\text{ MoZn}}_{{0.{85}}} {\text{S}}_{{2}} /{\text{PANI}} $$

### Air-Rechargeable Mechanism

**Fig. 5:**
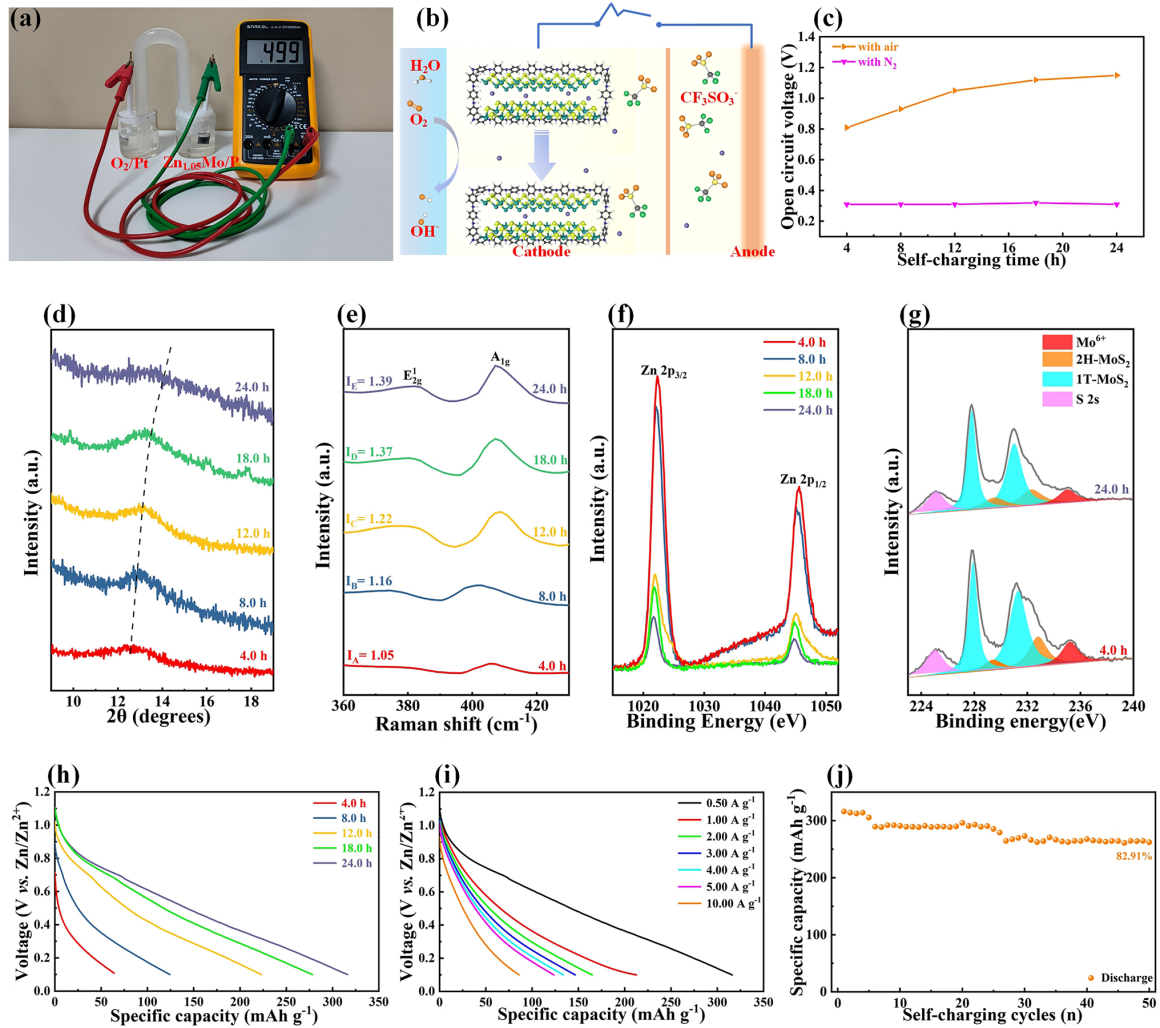
Mechanism of the redox reaction between Zn_0.85_Mo/P and O_2_ and air recharging/galvanostatic discharging behavior of Zn//Zn_0.85−*x*_Mo/P batteries. **a** Optical image of the designed galvanic cell. **b** Working mechanism of air-rechargeable ZIBs. **c** Effect of the oxidation time on OCV of Zn//Zn_0.85−*x*_Mo/P batteries. **d** XRD patterns, **e** Raman patterns, XPS spectra of **f** Zn and **g** Mo of Zn//Zn_0.85−*x*_Mo/P batteries after different air-recharging time. **h** Galvanostatic discharging curves of Zn//Zn_0.85−*x*_Mo/P batteries after different air-recharging time. **i** Galvanostatic discharging curves of Zn//Zn_0.85−*x*_Mo/P batteries at different current density after 24.0-h air recharging. **j** Air recharging/galvanostatic discharging life of Zn//Zn_0.85−*x*_MoS batteries

The MoS_2_/PANI cathode exhibits excellent Zn^2+^ storage performance, and the Mo was reduced/oxidized during the insertion/extraction of Zn^2+^. During electrochemical charging, electrons are released from the MoZn_0.85_S_2_/PANI (Zn_0.85_Mo/P) cathode and Mo in Zn_0.85_Mo/P was oxidized and Zn^2+^ was extracted from the layered structure. In this process, the driving force for Zn_0.85_Mo/P to release electrons is generally an external power supply. In addition to the above electrochemical oxidation reactions, other strategies that can realize electron transfer are also expected to be used in the charging process of air-rechargeable ZIBs based on Zn_0.85_Mo/P cathode. Among various oxidants, O_2_ is common and abundant in air and has the standard electrode potentials of ~ 0.40 and ~ 1.23 V *vs.* SHE in the neutral and acidic medium, respectively. Therefore, if the redox potential of Zn_0.85_Mo/P cathode is lower than O_2_, the oxidation of Mo and the extraction of Zn^2+^ of Zn_0.85_Mo/P cathode can be realized. In order to verify the spontaneity of the redox reaction between Zn_0.85_Mo/P and O_2_ in 2.0 M Zn(CF_3_SO_3_)_2_ electrolyte, a galvanic cell was designed. As shown in Fig. 5a, the Zn_0.85_Mo/P electrode served as anode with 2.0 M Zn(CF_3_SO_3_)_2_ electrolyte and was sealed under N_2_ to avoid the dissolvation of oxygen. The platinum sheet was used as cathode, which was immersed in 2.0 M Zn(CF_3_SO_3_)_2_ electrolyte containing dissolved oxygen. In this system, a cell voltage of 0.499 V was observed. According to the relationship between thermodynamic function and cell voltage:

6 $$ \Delta G = - nEF $$

where Δ*G*, *F*, *E* and *n* are the Gibbs free energy change, Faraday constant cell voltage and normal number, respectively, the Gibbs free energy change of above system is less than zero, indicating that the redox reaction between Zn_0.85_Mo/P and O_2_ can take place spontaneously in 2.0 M Zn(CF_3_SO_3_)_2_.The air-recharging mechanism of ZIBs based on Zn_0.85_Mo/P cathode is shown in Fig. 5b. Under the action of O^2^, Zn^2+^ is extracted from Zn_0.85_Mo/P, while O_2_ gains electrons from Zn_0.85_Mo/P and reacts with H_2_O to form OH^−^. The generated OH^−^ and extracted Zn^2+^ combine with the adsorbed electrolyte ions (Zn^2+^ and CF_3_SO_3_^2−^) to form an amorphous trifluoride containing Zn_*x*+*y*_(CF_3_SO_3_)_2*y*_(OH)_2*x*_ [Figs. S33-S35] [18]. Therefore, the fully discharged Zn_0.85_Mo/P cathode can be restored to the charged state Zn_0.85-x_Mo/P cathode through spontaneous redox reaction. Subsequently, the curve of open-circuit voltage (OCV) with and without O_2_ as a function of air-recharging time was tested (Fig. 5c), which further prove that O_2_ plays an important role. More importantly, it is found that after 24.0 h of oxidation, the OCV of the ZIBs reached 1.15 V, close to the initial OCV (1.21 V). In order to demonstrate the above mechanisms, ex situ characterization was carried out to study the structure and composition evolution of Zn_0.85-*x*_Mo/P electrodes. As the air-rechargeable time is extended from 0 to 24.0 h, the XRD peak (002) of Zn_0.85-x_Mo/P electrodes gradually shifts to a large angle (Fig. 5d), and the intensity ratio of *A*_1g_/*E*^1^_2g_ in Raman spectrum increases from 1.05 to 1.39 (Fig. 5e). This result is similar to that of chemical charging, indicating that Zn^2+^ gradually extracted from Zn_0.85-x_Mo/P electrode. In order to further prove the air-recharging mechanism of Zn_0.85-x_Mo/P electrode, XPS was carried out. With the increases of air-recharging time, the content of Zn in Zn_0.85-*x*_Mo/P electrode gradually decreased (Fig. 5f) and the Mo 3*d* peak gradually moved to a higher binding energy (Fig. 5g), indicating the gradual extraction of Zn^2+^ and oxidation of Mo in Zn_0.85-*x*_Mo/P cathode during the air-recharging process. The above discussion proves that the air-rechargeable ZIB based on Zn_0.85-*x*_Mo/P cathode can be charged by O_2_. In order to determine the air-recharging performance of ZIB based on Zn_0.85-*x*_Mo/P cathode, the galvanostatic current discharge curves after air recharging for different times were tested (Fig. 5h). Since the air-rechargeable battery needs to consume zinc electrodes continuously during its operation, the mass of the zinc anode is strictly controlled at 21.2 mg to assemble the battery for practicability. After air recharging for 4.0 h, the corresponding discharge capacity of the ZIB can reach to 64.17 mAh g^−1^ at 0.50 A g^−1^. As the air-recharging time increases, the capacity of the ZIB gradually increases. 35 Rechargeable ZIB could provide 316.09, 212.69, 164.94, 146.50, 133.61, 123.47 and 85.83 mAh g^−1^ at the current densities of 0.50, 1.00, 2.00, 3.00, 4.00, 5.00 and 10.00 A g^−1^, respectively. As shown in Fig. S36, the air-rechargeable ZIB manifests high energy densities over a wider power density range which has the power density and energy density as high as 288.29 W kg^−1^ and 12.69 Wh kg^−1^, respectively. The stability of this air recharging/galvanostatic current discharge cycle was subsequently verified. In this test, the air-recharging ZIB based on Zn_0.85-*x*_Mo/P cathode was discharged to 0.10 V at a current density of 0.50 A g^−1^ and then air recharged for 24.0 h and galvanostatic discharge at 0.50 A g^−1^. As shown in Fig. 5j, the air-rechargeable ZIB can be cycled for 50 times. Afterward, the air-rechargeable ZIB shows a high discharge capacity of 291.22 mAh g^−1^ with the high capacity retention rate of 82.91%. Based on the above discussion, the redox reaction between Zn_0.85_Mo/P and O_2_ can be summarized as below:

7 $$ \begin{aligned}&{\text{MoZn}}_{{0.{85}}} {\text{S}}_{{2}} /{\text{PANI }} + \, 0.{5}x{\text{O}}_{{2}} + \, x{\text{H}}_{{2}} {\text{O }} + \, y{\text{Zn}}\left( {{\text{CF}}_{{3}} {\text{SO}}_{{3}} } \right)_{{2}} \to {\text{MoZn}}_{{0.{85} - x}} {\text{S}}_{{2}} /{\text{PANI }} \\& + {\text{ Zn}}_{x + y} \left( {{\text{CF}}_{{3}} {\text{SO}}_{{3}} } \right)_{{{2}y}} \left( {{\text{OH}}} \right)_{{{2}x}} \end{aligned}$$

### Practicability and Zn Batteries Module

**Fig. 6:**
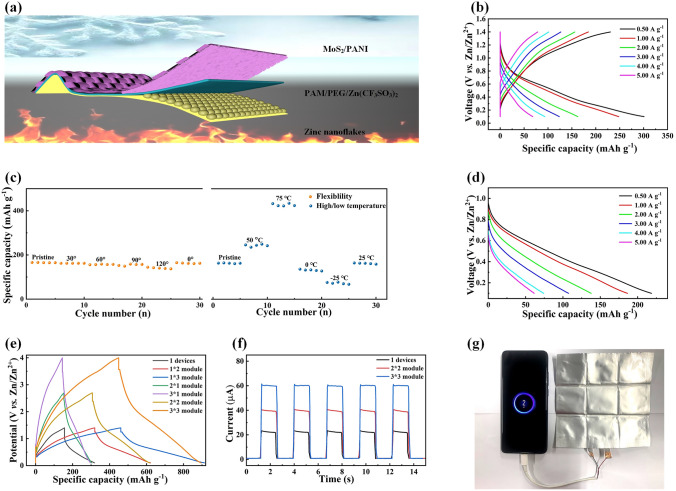
Practicability of MoS_2_/PANI cathode. **a** Schematic diagram and **b** GCD curves of the QSZIBs. **c** Specific capacity at 2.00 A g^−1^ of QSZIBs under bent or high and low temperatures. **d** Galvanostatic discharging curves of QSZIBs after 24.0-h air recharging. **e** GCD curves at 2.0 A g^−1^ of QSZIB module. The QSZIB module powered for **f** pressure sensors and **g** smartphones

In order to illustrate the practicability of MoS_2_/PANI cathode, QSZIBs were assembled by MoS_2_/PANI cathode, zinc nanoflakes synthesized on carbon cloth by electrodeposition (Fig. S37) to assemble the battery for practicability. Anode and PAM/PEG/Zn(CF_3_SO_3_)_2_ served as hydrogel electrolyte (Fig. 6a). As shown in Fig. 6b, the QSZIB shows excellent electrochemical performance. The discharge capacities at 0.50, 1.00, 2.00, 3.00, 4.00 and 5.00 A g^−1^ are 300.96, 248.04, 162.47, 123.63, 93.44 and 68.06 mAh g^−1^, respectively. Considering that batteries often need to be operated in extreme conditions, QSZIB was tests under bent or high and low temperatures (Fig. 6c). The QSZIB shows only slight capacity variation under different bending states, indicating the excellent flexibility of the battery. Even more impressively, the QSZIB demonstrated high capacity and stability at both high and low temperature, respectively. Subsequently, the air-recharging ability of QSZIB was verified. After air recharging for 24.0 h, the fully discharged QSZIB shows a high capacity at different current densities (Fig. 6d). Considering the low capacity and voltage of QSZIB caused by the inherent characteristics of water batteries, a 3 × 3 module was constructed to further verify the practicality (Fig. 6e). In subsequent tests, the module was found to successfully power pressure sensors (Fig. 6f) and smartphones (Fig. 6g), showing great practical prospects.

## Conclusions

In summary, we design a air-rechargeable ZIB based on MoS_2_/PANI cathode. The introduction of conductive polymer (PANI) coating of MoS_2_ not only improves the conductivity, but also induces the charge redistribution and structure changes at the interface to weaken the electrostatic interaction, thus promoting the diffusion of Zn^2+^. Accordingly, MoS_2_/PANI cathodes show the best electrochemical performance of zinc ion storage among the Mo-based cathodes so far. Benefited from the low reduction potential of the MoS_2_/PANI cathodes (0.71 V vs*.* Zn/Zn^2+^), the ZIBs can be quickly and deeply air recharged by oxygen (316.09 mAh g^−1^ at 0.50 A g^−1^ after 24-h air recharge, *i.e.,* 89.99% capacity retain of galvanostatic charge at 0.50 A g^−1^) and have long air recharging/galvanostatic discharging life (50 cycles). As a proof of concept, the quasi-solid-state ZIBs employing MoS_2_/PANI cathode and PAM/PEG/Zn(CF_3_SO_3_)_2_ demonstrate great electrochemical performance, excellent flexibility, high- and low-temperature stability and air-rechargeable ability. Finally, to verify the practicality of the battery, a 3 × 3 battery module was successfully assembled to power a pressure sensor and a smartphone. This work will provide a promising research direction for the material design and device testing of the next-generation self-powered system.

### Supplementary Information

Below is the link to the electronic supplementary material.

Supplementary file1 (PDF 2456 KB)

## References

[1] Zichittella G, Perez-Ramirez J (2021). Status and prospects of the decentralised valorisation of natural gas into energy and energy carriers. Chem. Soc. Rev..

[2] Veers P, Dykes K, Lantz E, Barth S, Bottasso CL (2019). Grand challenges in the science of wind energy. Science.

[3] Li Z, Gao Y, Zhang Z, Xiong Q, Deng L (2021). cPCN-regulated SnO_2_ composites enables perovskite solar cell with efficiency beyond 23%. Nano-Micro Lett..

[4] Zhang N, Huang F, Zhao S, Lv X, Zhou Y (2020). Photo-rechargeable fabrics as sustainable and robust power sources for wearable bioelectronics. Matter.

[5] Peng P, Zhou J, Liang L, Huang X, Lv H (2022). Regulating thermogalvanic effect and mechanical robustness via redox ions for flexible quasi-solid-state thermocells. Nano-Micro Lett..

[6] Li Z, Xu Y, Wu L, An Y, Sun Y (2022). Zinc ion thermal charging cell for low-grade heat conversion and energy storage. Nat. Commun..

[7] Zhang Q, Liang Q, Nandakumar DK, Qu H, Shi Q (2021). Shadow enhanced self-charging power system for wave and solar energy harvesting from the ocean. Nat. Commun..

[8] Zhao K, Wang Y, Han L, Wang Y, Luo X (2019). Nanogenerator-based self-charging energy storage devices. Nano-Micro Lett..

[9] Chen P, Li TT, Yang YB, Li GR, Gao XP (2022). Coupling aqueous zinc batteries and perovskite solar cells for simultaneous energy harvest, conversion and storage. Nat. Commun..

[10] Wang X, Huang YT, Liu C, Mu K, Li KH (2019). Direct thermal charging cell for converting low-grade heat to electricity. Nat. Commun..

[11] Conta G, Libanori A, Tat T, Chen G, Chen J (2021). Triboelectric nanogenerators for therapeutic electrical stimulation. Adv. Mater..

[12] Liu T, Wang J, Xu Y, Zhang Y, Wang Y (2021). Dendrite-free and stable lithium metal battery achieved by a model of stepwise lithium deposition and stripping. Nano-Micro Lett..

[13] He H, Zhang H, Huang D, Kuang W, Li X (2022). Harnessing plasma-assisted doping engineering to stabilize metallic phase MoSe_2_ for fast and durable sodium-ion storage. Adv. Mater..

[14] Xiong Q, He H, Zhang M (2022). Design of flexible films based on kinked carbon nanofibers for high rate and stable potassium-ion storage. Nano-Micro Lett..

[15] Zhao L, Liu Z, Chen D, Liu F, Yang Z (2021). Laser synthesis and microfabrication of micro/nanostructured materials toward energy conversion and storage. Nano-Micro Lett..

[16] Gao Y, Pan Z, Sun J, Liu Z, Wang J (2022). High-energy batteries: beyond lithium-ion and their long road to commercialisation. Nano-Micro Lett..

[17] Li X, Ma Y, Yue Y, Li G, Zhang C (2022). A flexible Zn-ion hybrid micro-supercapacitor based on MXene anode and V_2_O_5_ cathode with high capacitance. Chem. Eng. J..

[18] Lin D, Li Y (2022). Recent advances of aqueous rechargeable zinc-iodine batteries: challenges, solutions, and prospects. Adv. Mater..

[19] Zhao S, Luo X, Cheng Y, Shi Z, Huang T (2023). A flexible zinc ion hybrid capacitor integrated system with layers-dependent V_2_CT_x_ MXene. Chem. Eng. J..

[20] Adekoya D, Qian S, Gu X, Wen W, Li D (2020). DFT-guided design and fabrication of carbon-nitride-based materials for energy storage devices: a review. Nano-Micro Lett..

[21] Chen K, Kim S, Je M, Choi H, Shi Z (2021). Ultrasonic plasma engineering toward facile synthesis of single-atom M-N_4_/N-doped carbon (M = Fe, Co) as superior oxygen electrocatalyst in rechargeable zinc-air batteries. Nano-Micro Lett..

[22] Dong F, Wu M, Chen Z, Liu X, Zhang G (2021). Atomically dispersed transition metal-nitrogen-carbon bifunctional oxygen electrocatalysts for zinc-air batteries: recent advances and future perspectives. Nano-Micro Lett..

[23] Niu Y, Teng X, Gong S, Xu M, Sun SG (2021). Engineering two-phase bifunctional oxygen electrocatalysts with tunable and synergetic components for flexible Zn-air batteries. Nano-Micro Lett..

[24] Zhang Y, Wan F, Huang S, Wang S, Niu Z (2020). A chemically self-charging aqueous zinc-ion battery. Nat. Commun..

[25] Yan L, Zhang Y, Ni Z, Zhang Y, Xu J (2021). Chemically self-charging aqueous zinc-organic battery. J. Am. Chem. Soc..

[26] Li W, Han C, Gu Q, Chou SL, Wang JZ (2020). Electron delocalization and dissolution-restraint in vanadium oxide superlattices to boost electrochemical performance of aqueous zinc-ion batteries. Adv. Energy Mater..

[27] Liu S, Zhu H, Zhang B, Li G, Zhu H (2020). Tuning the kinetics of zinc-ion insertion/extraction in V_2_O_5_ by in situ polyaniline intercalation enables improved aqueous zinc-ion storage performance. Adv. Mater..

[28] Huang J, Wang Z, Hou M, Dong X, Liu Y (2018). Polyaniline-intercalated manganese dioxide nanolayers as a high-performance cathode material for an aqueous zinc-ion battery. Nat. Commun..

[29] Ma X, Cao X, Yao M, Shan L, Shi X (2022). Organic-inorganic hybrid cathode with dual energy-storage mechanism for ultrahigh-rate and ultralong-life aqueous zinc-ion batteries. Adv. Mater..

[30] Yao Z, Wu Q, Chen K, Liu J, Li C (2020). Shallow-layer pillaring of a conductive polymer in monolithic grains to drive superior zinc storage via a cascading effect. Energy Environ. Sci..

[31] Bin D, Huo W, Yuan Y, Huang J, Liu Y (2020). Organic-inorganic-induced polymer intercalation into layered composites for aqueous zinc-ion battery. Chem.

[32] Bi W, Gao G, Wu G, Atif M, AlSalhi MS (2021). Sodium vanadate/PEDOT nanocables rich with oxygen vacancies for high energy conversion efficiency zinc ion batteries. Energy Storage Mater..

[33] Zhang Z, Xi B, Wang X, Ma X, Chen W (2021). Oxygen defects engineering of VO_2_·xH_2_O nanosheets via in situ polypyrrole polymerization for efficient aqueous zinc ion storage. Adv. Funct. Mater..

[34] Hu L, Wu Z, Lu C, Ye F, Liu Q (2021). Principles of interlayer-spacing regulation of layered vanadium phosphates for superior zinc-ion batteries. Energy Environ. Sci..

[35] Li S, Liu Y, Zhao X, Cui K, Shen Q (2021). Molecular engineering on MoS_2_ enables large interlayers and unlocked basal planes for high-performance aqueous Zn-ion storage. Angew. Chem. Int. Ed..

[36] Liang H, Cao Z, Ming F, Zhang W, Anjum DH (2019). Aqueous zinc-ion storage in MoS_2_ by tuning the intercalation energy. Nano Lett..

[37] Liu J, Xu P, Liang J, Liu H, Peng W (2020). Boosting aqueous zinc-ion storage in MoS_2_ via controllable phase. Chem. Eng. J..

[38] Liu H, Wang JG, Hua W, You Z, Hou Z (2021). Boosting zinc-ion intercalation in hydrated MoS_2_ nanosheets toward substantially improved performance. Energy Storage Mater..

[39] Xu W, Sun C, Zhao K, Cheng X, Rawal S (2019). Defect engineering activating (boosting) zinc storage capacity of MoS_2_. Energy Storage Mater..

[40] Li H, Yang Q, Mo F, Liang G, Liu Z (2019). MoS_2_ nanosheets with expanded interlayer spacing for rechargeable aqueous Zn-ion batteries. Energy Storage Mater..

[41] Li S, Liu Y, Zhao X, Shen Q, Zhao W (2021). Sandwich-like heterostructures of MoS_2_/graphene with enlarged interlayer spacing and enhanced hydrophilicity as high-performance cathodes for aqueous zinc-ion batteries. Adv. Mater..

[42] Li C, Liu C, Wang Y, Lu Y, Zhu L (2022). Drastically-enlarged interlayer-spacing MoS_2_ nanocages by inserted carbon motifs as high performance cathodes for aqueous zinc-ion batteries. Energy Storage Mater..

[43] Jiang J, Chen Z, Hu Y, Xiang Y, Zhang L (2021). Flexo-photovoltaic effect in MoS_2_. Nat. Nanotechnol..

[44] Zhang X, Liao Q, Liu S, Kang Z, Zhang Z (2017). Poly(4-styrenesulfonate)-induced sulfur vacancy self-healing strategy for monolayer MoS_2_ homojunction photodiode. Nat. Commun..

[45] Zhang W, Liu Y, Pei X, Yuan Z, Zhang Y (2022). Stretchable MoS_2_ artificial photoreceptors for e-skin. Adv. Funct. Mater..

[46] Shi J, Wang S, Chen X, Chen Z, Du X (2019). An ultrahigh energy density quasi-solid-state zinc ion microbattery with excellent flexibility and thermostability. Adv. Energy Mater..

[47] Shi J, Wang S, Wang Q, Chen X, Du X (2020). A new flexible zinc-ion capacitor based on δ-MnO_2_@carbon cloth battery-type cathode and MXene@cotton cloth capacitor-type anode. J. Power Sour..

[48] Cui F, Wang D, Hu F, Yu X, Guan C (2022). Deficiency and surface engineering boosting electronic and ionic kinetics in NH_4_V_4_O_10_ for high-performance aqueous zinc-ion battery. Energy Storage Mater..

[49] Shi J, Hou Y, Liu Z, Zheng Y, Wen L (2022). The high-performance MoO_3−x_/MXene cathodes for zinc-ion batteries based on oxygen vacancies and electrolyte engineering. Nano Energy.

[50] Zheng P, Li T, Chi K, Xiao C, Fan J (2019). DFT insights into the formation of sulfur vacancies over corner/edge site of Co/Ni-promoted MoS_2_ and WS_2_ under the hydrodesulfurization conditions. Appl. Catal. B Environ..

[51] Tian Y, Xu L, Li M, Yuan D, Liu X (2020). Interface engineering of CoS/CoO@N-doped graphene nanocomposite for high-performance rechargeable Zn-air batteries. Nano-Micro Lett..

[52] Wagh NK, Shinde SS, Lee CH, Kim SH, Kim DH (2022). Supramolecular polymer intertwined free-standing bifunctional membrane catalysts for all-temperature flexible Zn-air batteries. Nano-Micro Lett..

[53] Lee WSV, Xiong T, Wang X, Xue J (2021). Unraveling MoS_2_ and transition metal dichalcogenides as functional zinc-ion battery cathode: a perspective. Small Methods.

[54] Zhang H, Wu W, Liu Q, Yang F, Shi X (2021). Interlayer engineering of alpha-MoO_3_ modulates selective hydronium intercalation in neutral aqueous electrolyte. Angew. Chem. Int. Ed..

[55] Xue Y, Guo Y, Zhang Q, Xie Z, Wei J (2022). MOF-derived Co and Fe species loaded on N-doped carbon networks as efficient oxygen electrocatalysts for Zn-air batteries. Nano-Micro Lett..

[56] Zhu Y, Yue K, Xia C, Zaman S, Yang H (2021). Recent advances on MOF derivatives for non-noble metal oxygen electrocatalysts in zinc-air batteries. Nano-Micro Lett..

[57] Jain T, Rasera BC, Guerrero RJS, Boutilier MSH, O'Hern SC (2015). Heterogeneous sub-continuum ionic transport in statistically isolated graphene nanopores. Nat. Nanotechnol..

[58] Xin W, Fu J, Qian Y, Fu L, Kong XY (2022). Biomimetic KcsA channels with ultra-selective K^+^ transport for monovalent ion sieving. Nat. Commun..

