# Supplementary material for: An Air-Rechargeable Zn Battery Enabled by Organic–Inorganic Hybrid Cathode
Source: Nanomicro Lett. 2023 Feb 16;15:53. doi: 10.1007/s40820-023-01023-7 (PMC9935787; doi:10.1007/s40820-023-01023-7)
Supplement: Supplementary file 1 — Supplementary file1 (PDF 2456 KB) [file 40820_2023_1023_MOESM1_ESM.pdf]

Supporting Information

An Air-Rechargeable Zn Battery Enabled by Organic–Inorganic Hybrid Cathode

Junjie Shi[1,#], Ke Mao[1,2,#], Qixiang Zhang[1], Zunyu Liu[1], Fei Long[1], Li Wen[1], Yixin Hou[1], Xinliang Li[3], Yanan Ma[4], Yang Yue[5,*], Luying Li[1], Chunyi Zhi[3], Yihua Gao[1,4,*]

[1] Center for Nanoscale Characterization & Devices (CNCD), Wuhan National Laboratory for Optoelectronics (WNLO) and School of Physics, Huazhong University of Science and Technology (HUST), Wuhan 430074, P. R. China

[2] College of Materials Science and Engineering, Guangxi Key Laboratory of Optical and Electronic Materials and Devices, Guilin University of Technology, Guilin 541004, P. R. China

[3] Hong Kong Center for Cerebro-Cardiovascular Health Engineering, Hong Kong SAR 999077, P. R. China

[4] Hubei Key Laboratory of Critical Materials of New Energy Vehicles & School of Mathematics, Physics and Optoelectronic Engineering, Hubei University of Automotive Technology, Shiyan 442002, P. R. China

[5] Information Materials and Intelligent Sensing Laboratory of Anhui Province, Key Laboratory of Structure and Functional Regulation of Hybrid Materials of Ministry of Education, Institutes of Physical Science and Information Technology, Anhui University, Hefei 230601, P. R. China

[#] Junjie Shi and Ke Mao contributed equally to this work.

*Corresponding authors. E-mail: yueyang@ahu.edu.cn (Yang Yue); gaoyihua@hust.edu.cn (Yihua Gao)

S1 Experiments Section

S1.1 Reagents and Materials

Zinc sulfate heptahydrate ($ZnSO_4 \cdot 7H_2O$), thiourea, ammonium molybdate tetrahydrate ($(NH_4)_6Mo_7O_{24} \cdot 4H_2O$), aniline and zinc trifluoromethanesulfonate ($Zn(CF_3SO_3)_2$) were purchased from Aladdin Reagents (Shanghai) Co., Ltd. Hydrochloric acid (HCl), ammonium persulfate ($(NH_4)_2S_2O_8$), acrylamide, $K_2S_2O_8$ and N, N′-methylenebisacrylamide were purchased from Sinopharm Chemical Reagent Co., Ltd. All the above reagents are analytically pure and do not require further purification.

S1.2 Synthesis of Pristine PANI and MoS_2

0.30 mL aniline and 0.180 g $(NH_4)_2S_2O_8$ was dissolved in 12.0 mL HCl solution (1.0

mol L^{-1}), respectively. The two solutions were mixed together under strong stirring, then kept in an ice bath for 12.0 h. The resulting precipitation was collected and washed with deionized water for three times, and dried at 70 °C in a vacuum oven.

1.421 g thiourea and 0.724 g $(NH_4)_6Mo_7O_{24}\cdot4H_2O$ were dissolved in deionized water (21.8 mL) and stirred vigorously for 30.0 min to get a homogeneous solution. After the mixture was transferred to a Teflon-lined stainless-steel autoclave, it was heated to 180 °C and kept for 24.0 h. The resulting product was filtered, washed several times by deionized water and ethanol, and finally dried at 70 °C in a vacuum oven.

S1.3 Synthesis of MoS_2/PANI

1.0 mmol MoS_2 and 5.0 mg PANI were added in 24.0 mL HCl solution (1.0 mol L^{-1}). The solution was transferred into a plastic tube for probe sonication and the typical probe sonication lasted for 10.0 min. After the mixture was transferred to a Teflon-lined stainless-steel autoclave. Then, it was heated to 120 °C and kept for 18.0 h. The resulting precipitation was collected and washed with deionized water for three times, and dried at 70 °C in a vacuum oven. For comparison, MoS_2/PANI with more (10.0 mg) or less (1.0 mg) PANI addition was also studied.

S1.4 Preparation of Zinc Nanoflakes

Zinc nanoflakes were prepared by an electrodeposition method. Specifically, electrodeposition was carried out at −0.70 V (*vs*. Zn/Zn^{2+}) employing Zn metal plate as the reference and counter electrodes, carbon cloth as the working electrode, and 2.0 M $ZnSO_4$ solution as the electrolyte, respectively. The load of zinc nanosheet was about 5.0 mg cm^{-1}.

S1.5 Preparation of PAM-PEG Hydrogel

Firstly, 5.0 g acrylamide, 25.0 mg $K_2S_2O_8$ and 3.0 mg N, N′-methylenebisacrylamide were sequentially added to 30.0 ml deionized water. Then the mixture was stirred for 1.0 h and injected into a glass mould, followed by heating in an oven at 75 °C for 1.0 h. Finally, the as-fabricated hydrogel film was immersed in 2.0 M aqueous $Zn(CF_3SO_3)_2$ (The volume ratio of PEG-400 is 25.0%) solution for 12.0 h to achieve the equilibrated state.

S1.6 Materials Characterization

The morphology and microstructure of the products were characterized using scanning electron microscopy (SEM, FEI Nova NanoSEM 450) and transmission electron microscopy (TEM, FEI Titan G2 60-300). The structure and phase purity of the as-synthesized products were revealed by X-ray diffraction (XRD, Rigaku X-ray diffractometer with Cu-Kα radiation). X-ray photoelectron spectrometry (XPS) analysis was carried out on an AXIS-ULTRA DLD-600W spectrometer. Raman measurements were carried out on a LabRAM HR800. Thermogravimetric analysis (TGA) was conducted using an STA-449F3 instrument in an air atmosphere from 25 to 700 °C with a heating rate of 10 °C min^{-1}. ICP-OES/MS was carried out on a Hermo Fisher iCAP PRO (OES).

S1.7 Electrochemical Measurements

The electrode was prepared by mixing the active materials (20.0 mg), acetylene black (2.50 mg) and poly(vinylidene fluoride) (PVDF) (2.50 mg) in 500 μL *N*-methylpyrrolidinone (NMP) with a mass ratio of 8.0 : 1.0 : 1.0. The load of active material is about 1.0-1.4 mg cm^{-2}. 30 μm Zn metal foil was firstly washed with diluted hydrochloric acid for 3 min to remove the surficial oxide layer and then washed with deionized water and ethanol. The mass loading of cleaned Zn foil anode is about 21.2 mg cm^{-2}. Then, the obtained slurry was cast onto Ti foil and dried at 70 °C for 8.0 h. The electrochemical performances of electrodes and the quasi-solid zinc battery including cyclic voltammetry (CV), galvanostatic charge and discharge curves (GCD) and electrochemical impedance spectroscopy (EIS) were tested using the electrochemical workstation (AUT302N FRA2.V). The cycle life measurements and GITT were performed on a LAND-CT2001A battery workstation.

S1.8 Calculation Details

All the density functional theory (DFT) calculations were performed using the first principle plane-wave pseudopotential formulation as implemented in the Vienna Ab-initio Simulation Package (VASP) code. The generalized gradient approximation (GGA) within Perdew-Burke-Ernzerhof (PBE) was utilized as exchange correlation functional. The structures were relaxed until the forces were less than 0.010 eV/Å, and the energy convergent standard was 10^{-4} eV. The Monkhorst–Pack mesh of the supercell was set to 2×2×1. Climbing-image nudged elastic band (CI-NEB) method was used to calculate the activation barriers for Zn^{2+} hopping.

The discharge specific capacity was calculated from the results of galvanostatic discharge curves by the following Eq. S1:

$$C \frac{I*\Delta t}{m} \qquad \text{(S1)}$$

Where C is the specific mass capacity, I, Δt and m are the discharge current, discharge time and the mass of cathode active materials, respectively.

The energy density and power density are evaluated by the following Eqs. S2 and S3:

$$D_E = \int_0^{\Delta t} \frac{I*V(t)}{m} dt = \int_0^{\Delta Q} \frac{V(q)}{m} dq \qquad \text{(S2)}$$

$$D_P = \frac{D_E}{\Delta t} \qquad \text{(S3)}$$

Where D_E and D_P are respectively the energy density and power density, Δt and ΔQ are respectively the discharging time and discharge specific capacity, I is the discharge current, V is the working voltage, m is the mass of cathode active materials.

The capacity retention (CR) and coulomb efficiency (CE) are calculated by the following Eqs. S4 and S5:

$$CR = \frac{\Delta t}{\Delta t_0} \qquad (S4)$$

$$CE = \frac{\Delta t_d}{\Delta t_c} \qquad (S5)$$

Where Δt is the discharge time of different cycles and Δt_0 is the discharge time of the initial cycle. Δt_d is the discharge time and Δt_c is the charge time in the same cycle.

The battery was discharged or charged at 0.50 A g^{-1} for 3.0 min and then relaxed for 9.0 min to make the voltage reach equilibrium. The D_{Zn} can be calculated based on the simplified equation of $D_{Zn} = \frac{4}{\pi\tau}(\frac{m_B V_M}{M_B A})^2(\frac{\Delta E_S}{\Delta E_\tau})^2$, where m_B is the mass of the active material, M_B is the molecular weight, V_M is the molar volume, A is the total contact area between electrode and electrolyte, τ is the duration time of the current pulse, ΔE_τ is the variation of the battery voltage, and ΔE_S is related to the change of steady-state voltage for the corresponding step.

The *b* reflects the charge storage mechanism. *b* = 1/2 represents the diffusion control process while *b* =1 indicates the capacitive process. The $k_1 v$ and $k_2 v^{1/2}$ represent capacitive process and diffusion control process, respectively.

S2 Supplementary Figures and Tables

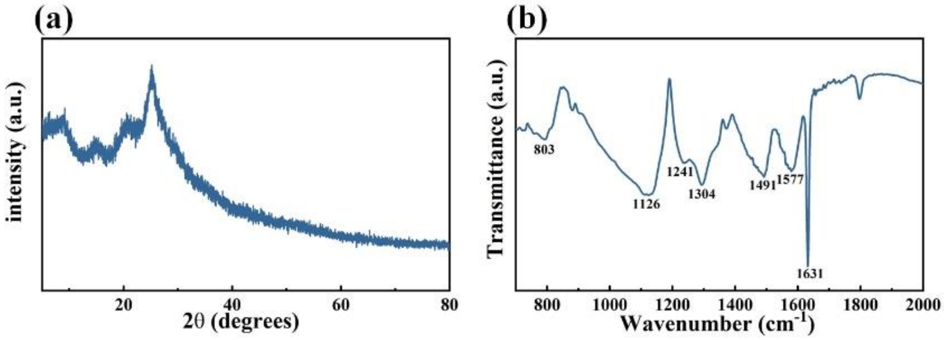

Fig. S1 a) XRD patterns and **b**) FTIR spectrum of PANI

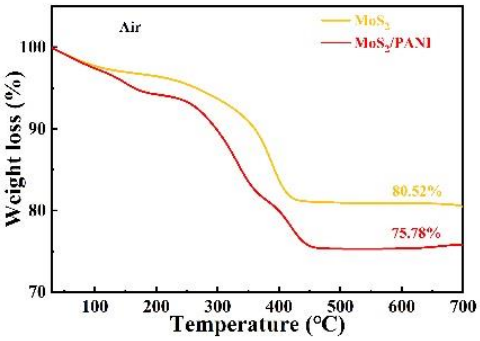

Fig. S2 TG curves of MoS_2 and MoS_2/PANI

The MoS_2 shows the 80.52% weight loss which is assigned to water evaporation and the oxidation of MoS_2 to MoO_3. It indicates the MoS_2 in total weight is ~ 89.47 wt%. MoS_2/PANI shows the total weight loss of 75.78 wt%, which indicates the MoS_2 in MoS_2/PANI is ~ 84.20 wt%. Therefore, the content of PANI in the MoS2/PANI can be calculated to be 5.27 wt%. In addition, the theoretical capacity of the MoS_2/PANI electrode was calculated. Under the ideal condition, MoS_2 will be transformed into Zn_2MoS_2 when completely discharged, indicating the theoretical capacity is 666.25 mAh g^{-1}. The proportion of PANI in MoS_2/PANI is 5.27%, so the theoretical capacity of MoS_2/PANI is 635.12 mAh g^{-1} without considering PANI energy storage.

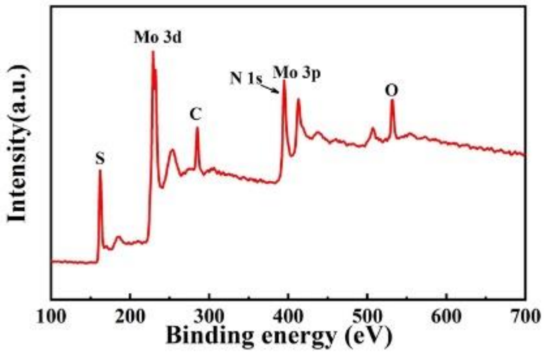

Fig. S3 Full XPS spectrum of MoS_2/PANI

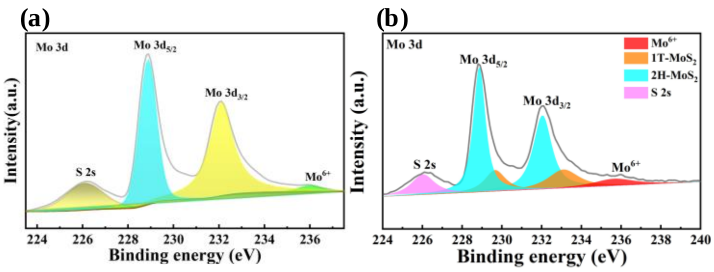

Fig. S4 a) Mo 3d XPS spectrum and **b**) fitted result related to phase composition of Mo 3d XPS spectra of the MoS_2/PANI

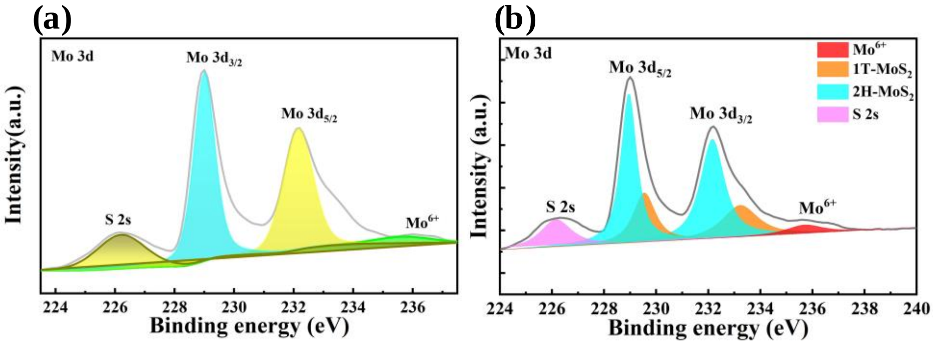

Fig. S5 a) Mo 3d XPS spectrum and **b**) fitted result related to phase composition of Mo 3d XPS spectra of the original MoS_2

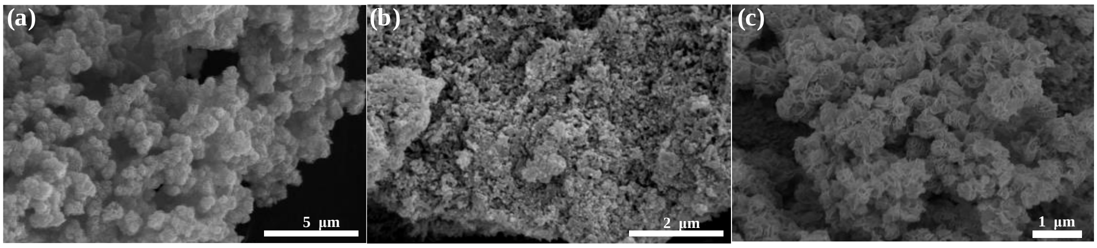

Fig. S6 SEM image of **a**) MoS_2, **b**) PANI and **c**) MoS_2/PANI

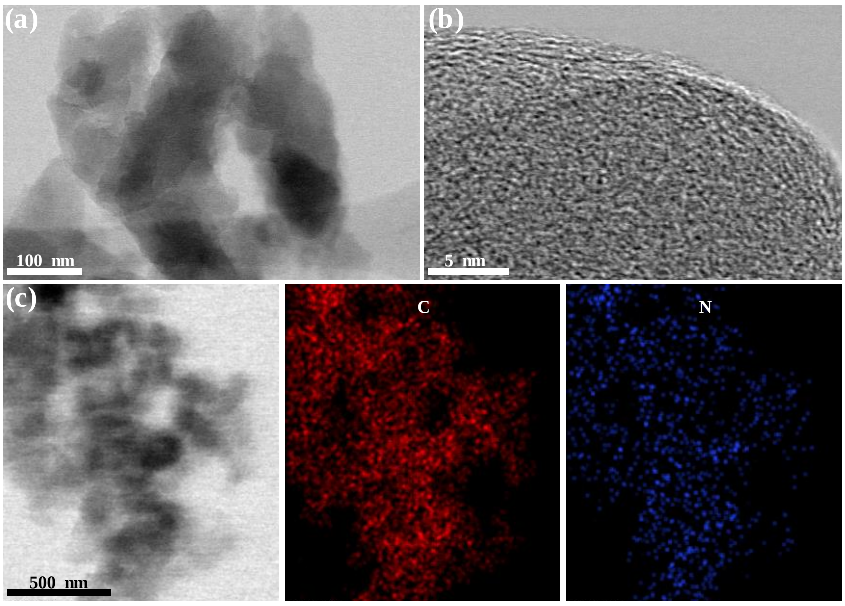

Fig. S7 a) TEM, **b**) HTEM and **c**) STEM elemental mapping image of PANI

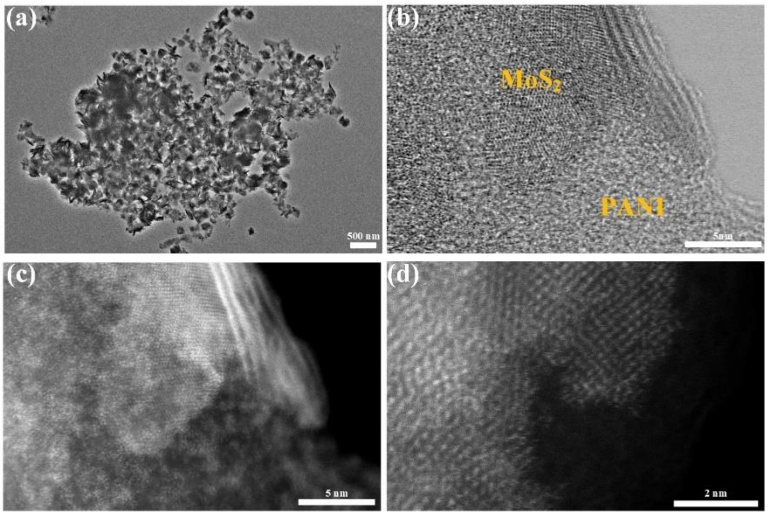

Fig. S8 a) TEM, **b**) HTEM, **c**) and **d**) HAADF-TEM image of MoS_2/PANI

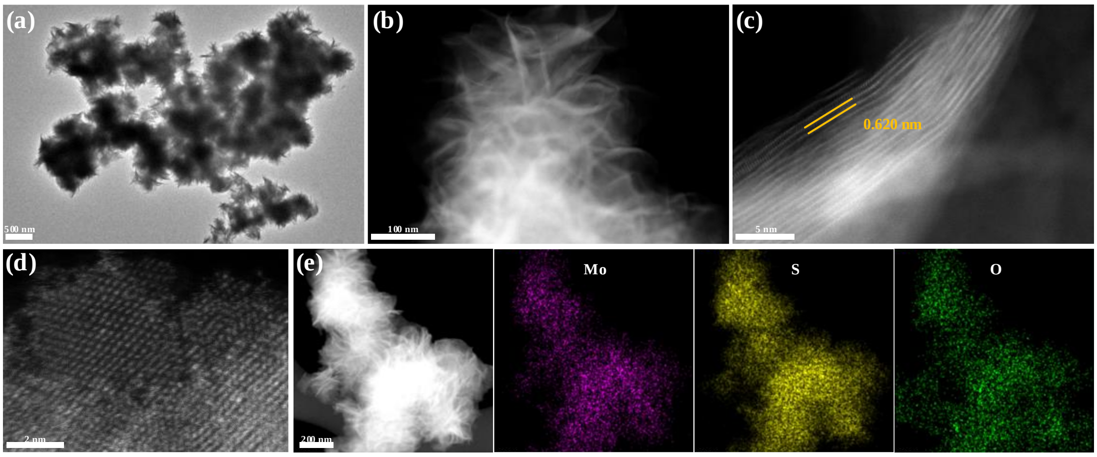

Fig. S9 a) TEM, **b, c**) HTEM, **d**) HAADF-TEM and **e**) STEM elemental mapping image of MoS_2. The STEM elemental mapping image in **Fig.** S9e reveal the homogeneous distribution of Mo, S and O elements along the MoS_2 nanoflowers

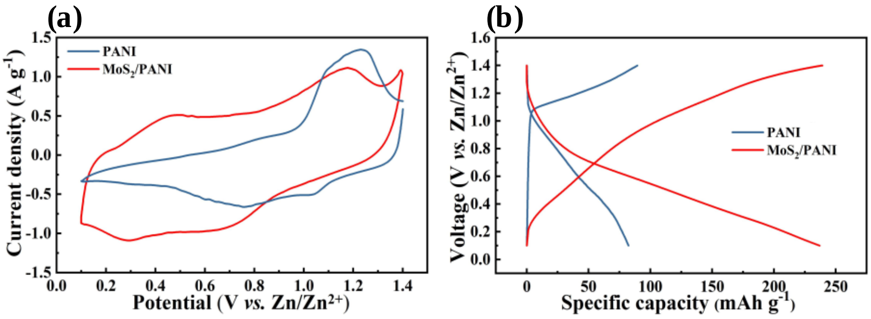

Fig. S10 a) CV curves at a scan rate of 1 mV s^{-1} and **b**) GCD curves at a current density of 2 A g^{-1} of the MoS_2/PANI and PANI

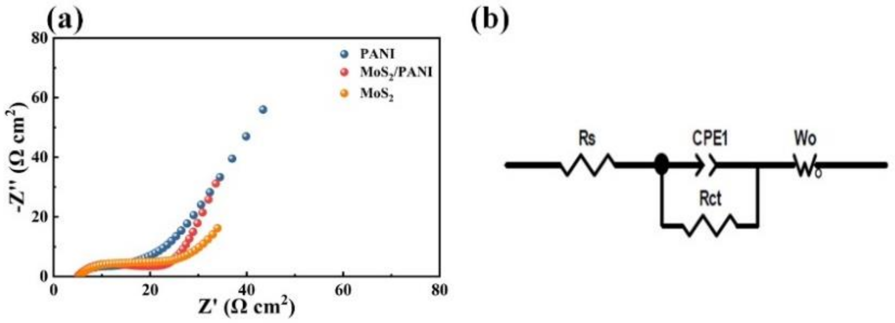

Fig. S11 a) Nyquist plot and **b**) equivalent circuit of MoS_2, PANI and MoS_2/PANI. The *R*s and *R*ct are internal resistance and charge transfer impedance, respectively

The electrochemical reaction kinetics of MoS_2, PANI and MoS_2/PANI were studied by electrical impedance spectroscopy (EIS). The resistance obtained from the equivalent circuit fit values is shown in Table S2. It is obvious that the PANI electrode shows the lowest intercept on the x-axis (internal resistance, *R*s) and the smallest semicircle (charge transfer impedance, *R*ct). The Rs denoting equivalent series resistance and Rct denoting charge transfer resistance. In addition, the *R*s and *R*ct of MoS_2/PANI are a medium value. These results can be understood by the facts that the proper coating of conductive PANI of MoS_2 can effectively increase the electrical conductivity and enhance electrochemical reaction kinetics.

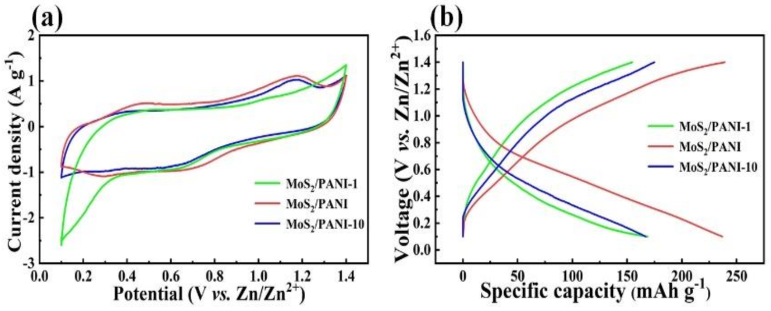

Fig. S12 a) CV curves at a scan rate of 1.0 mV s^{-1} and **b**) GCD curves at a current density of 2.0 A g^{-1} of the MoS_2/PANI cathodes with different amount of PANI

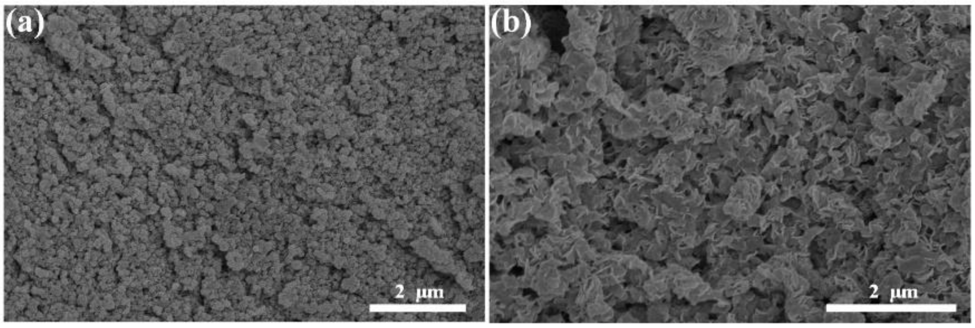

Fig. S13 SEM image of MoS_2/PANI with **a**) less and **b**) more amount of PANI

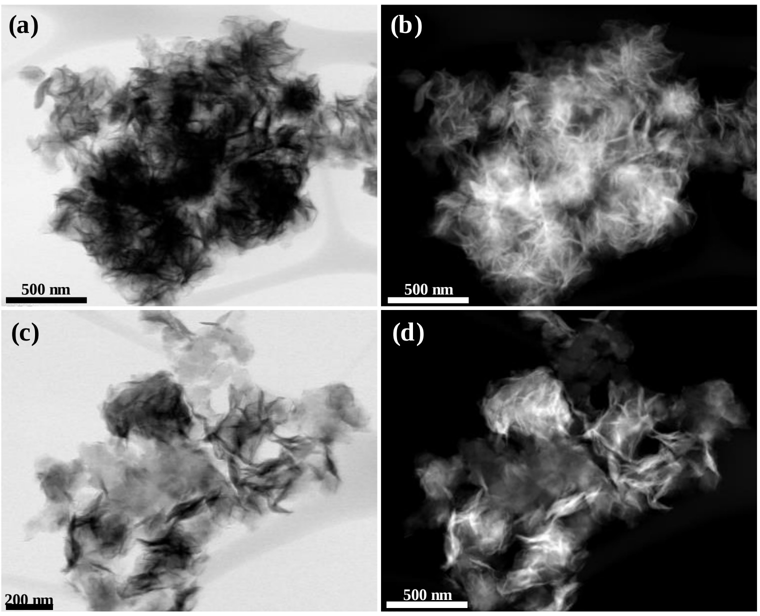

Fig. S14 TEM image of MoS_2/PANI with **a, b**) less and **c, d**) more amount of PANI. MoS_2/PANI with too little PANI still showed large and dense nanoflowers and MoS_2/PANI with too much PANI has an excess of PANI, which results in poor electrochemical performance.

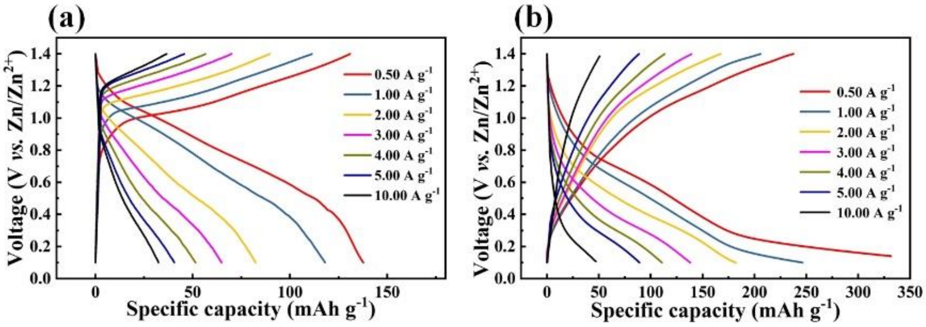

Fig. S15 The GCD curves of **a**) PANI and **b**) MoS_2 at current densities from 0.50 to 10.0 A g^{-1}

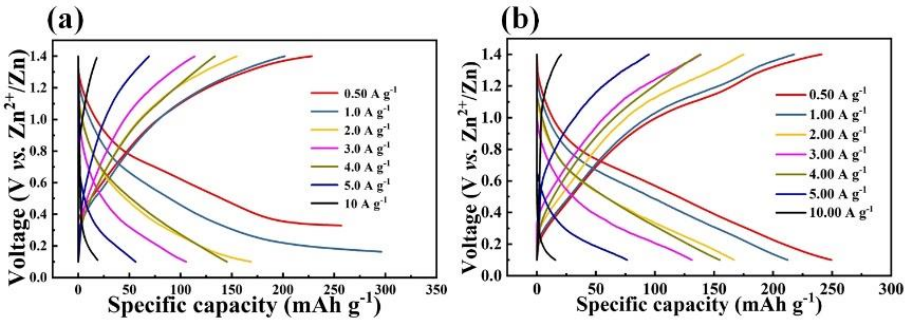

Fig. S16 GCD curves of MoS_2/PANI cathodes with different amount of PANI: **a**) less and **b**) more at current densities from 0.50 to 10.0 A g^{-1}

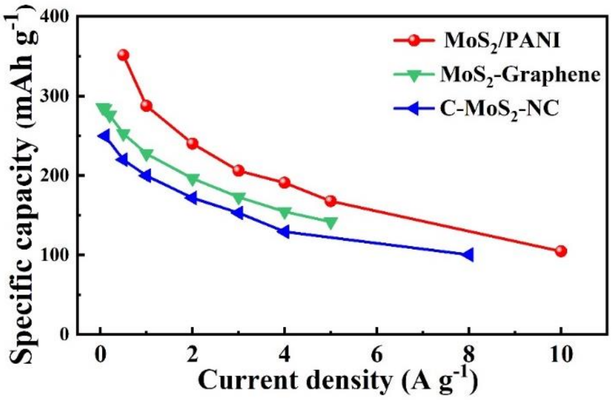

Fig. S17 The specific capacities comparison at different current density

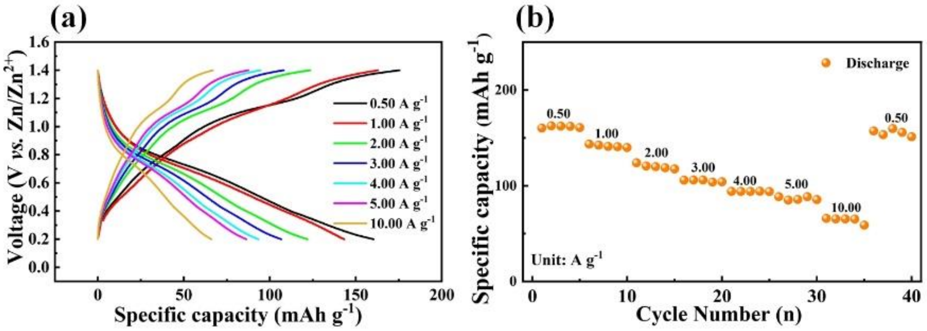

Fig. S18 GCD curves and rate capability of MoS_2 (0.2-1.4 V)

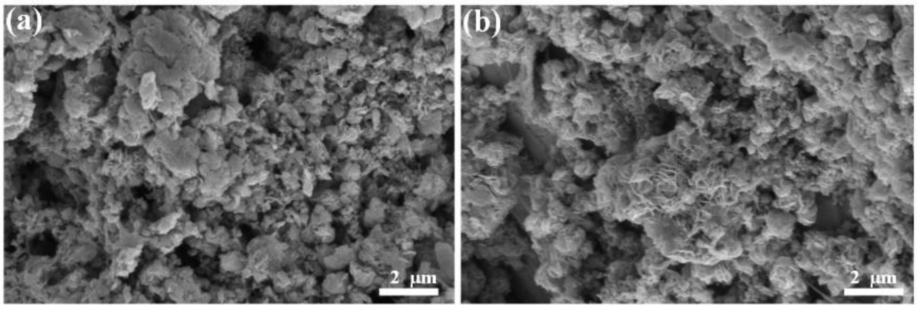

Fig. S19 The SEM image of **a**) original and **b**) after cycles charged to 1.40 V of MoS_2/PANI cathodes

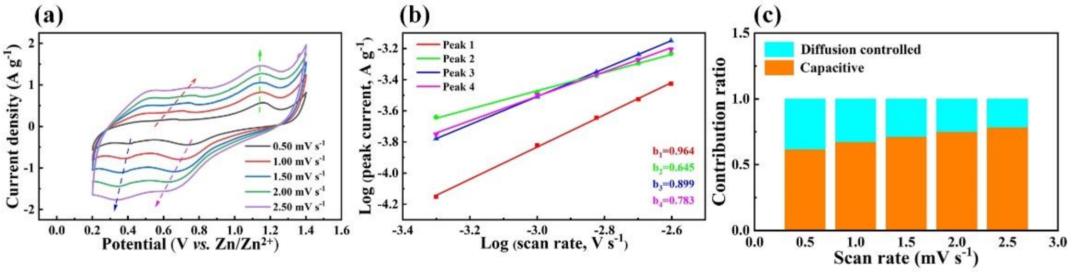

Fig. S20 a) CV curves at different scan rates from 0.50 to 2.50 mV s^{-1}, **b**) log (i) *vs.* log (v) plots and **c**) contribution ratios of diffusion-controlled and capacitive capacities at different scan rates of the MoS_2

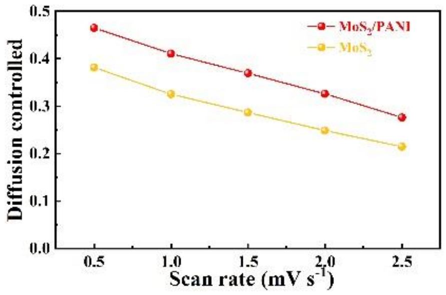

Fig. S21 The MoS_2 and MoS_2/PANI proportion of pseudocapacitance

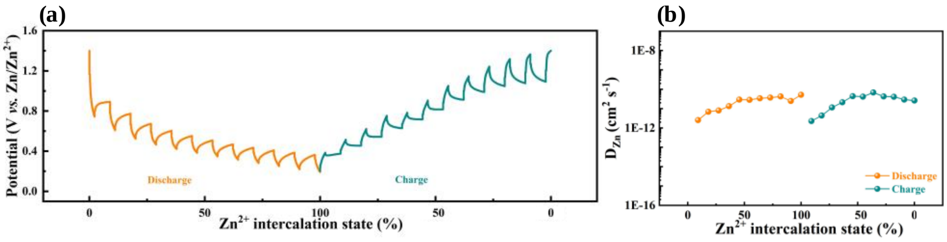

Fig. S22 a) GITT and **b**) D_{Zn} of MoS_2 cathode

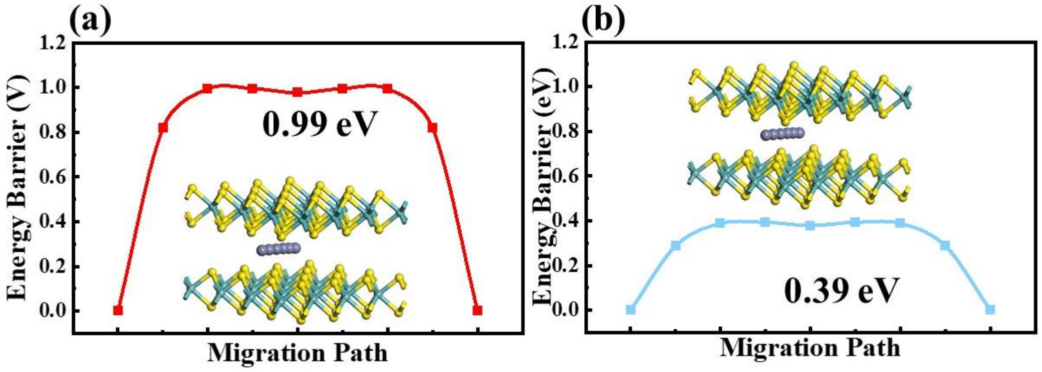

Fig. S23 DFT calculation of the diffusion path and migration barrier of Zn^{2+} in MoS_2 and MoS_2/PANI

The migration of zinc ions in MoS_2 layer is generally along the ab side from one octahedral (O) position to another octahedral position. Under normal circumstances, the layer spacing in MoS_2 is 0.620 nm. At this time, the migration barrier of zinc ions in the whole process will be large, 0.99 eV, due to the small layer spacing of MoS_2. Such A large barrier is not conducive to the rapid migration of zinc ions. When MoS_2 and PANI are combined, the layer spacing of MoS_2/PANI is enlarged to 0.682 nm, and the migration barrier of zinc ions is reduced to 0.39 eV. The large layer spacing greatly reduces the barrier of zinc ion migration and improves the conductivity of the electrode material. The Figure shows the diffusion path of Zn^{2+} and its migration barrier in MoS_2.

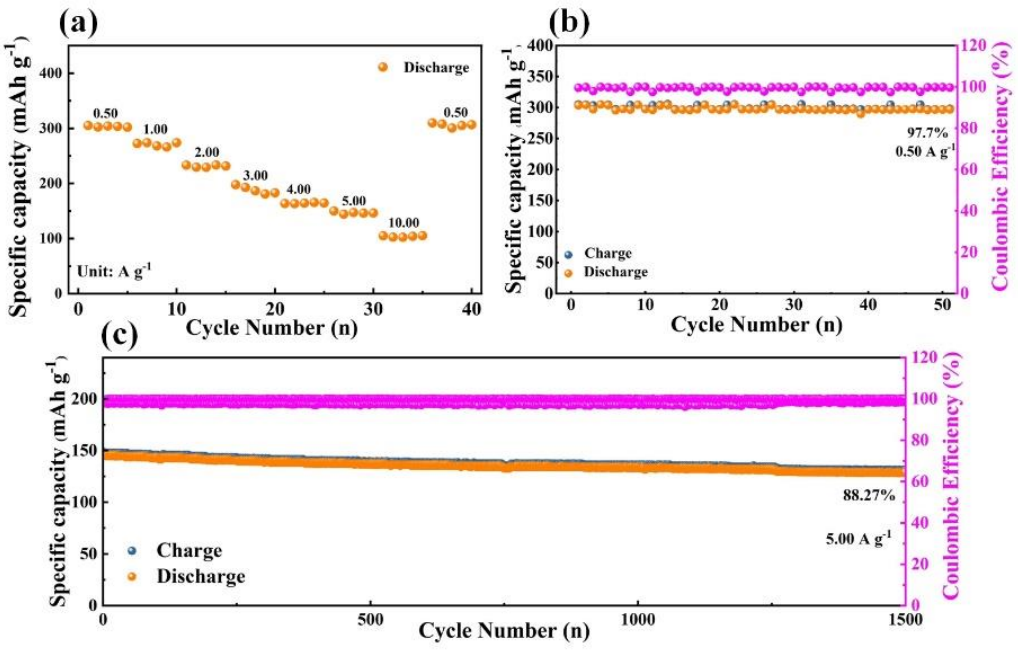

Fig. S24 a) The specific capacity, cyclic life **b**) at 0.50 A g^{-1} and **c**) 5.00 A g^{-1} of MoS_2/PANI cathode in N_2

Figure S24a shows the excellent rate capability of MoS_2/PANI cathode in N_2 (the specific capacity at 0.50, 1.00, 2.00, 3.00, 4.00, 5.00 and 10.00 A g^{-1} are 304.98, 272.56, 233.23, 197.78, 163.48, 159.43 and 104.44 mAh g^{-1}, respectively). In addition, the MoS_2/PANI cathode has higher cycle life and poorer specific capacity in N_2 due to air recharging capability.

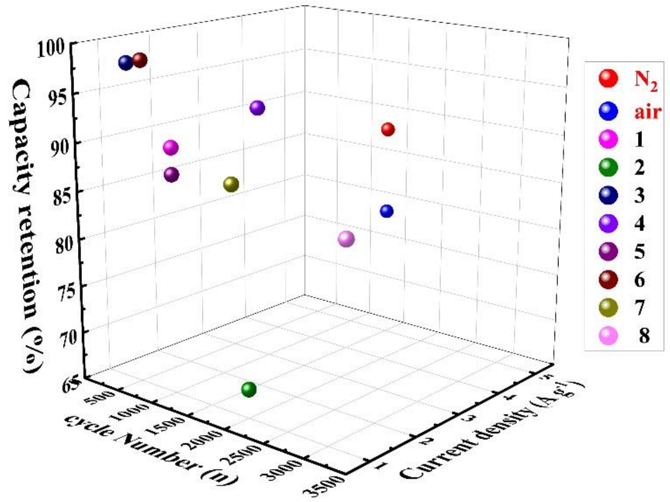

Fig. S25 The cycling performance of MoS_2/PANI cathode compare with those of the previously reported MoS_2 cathodes for ZIBs

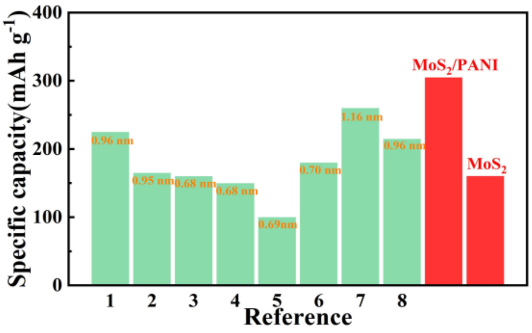

Fig. S26 The specific capacity and interlayer spacing of recent reported MoS_2-based cathodes and MoS_2/PANI cathodes at a current density of 0.50 A g^{-1}

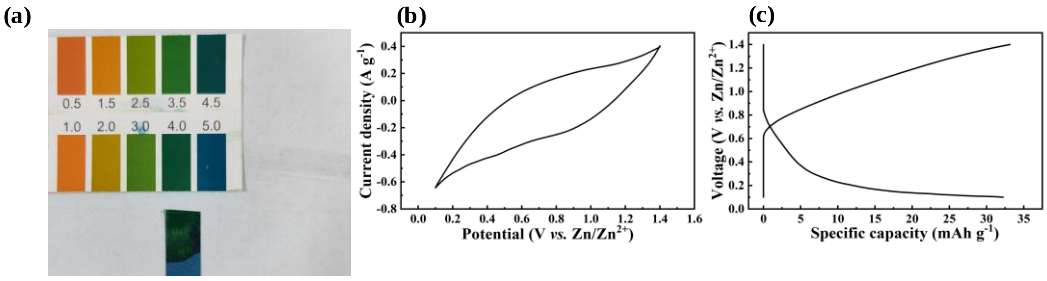

Fig. S27 a) PH Photo of 2.0 M $Zn(CF_3SO_3)_2$; **b**) CV curve at 1.00 mV s^{-1} and **c**) the specific capacity at a current density of 0.50 A g^{-1} of MoS_2/PANI cathodes in HCF_3SO_3 (pH=4.0)

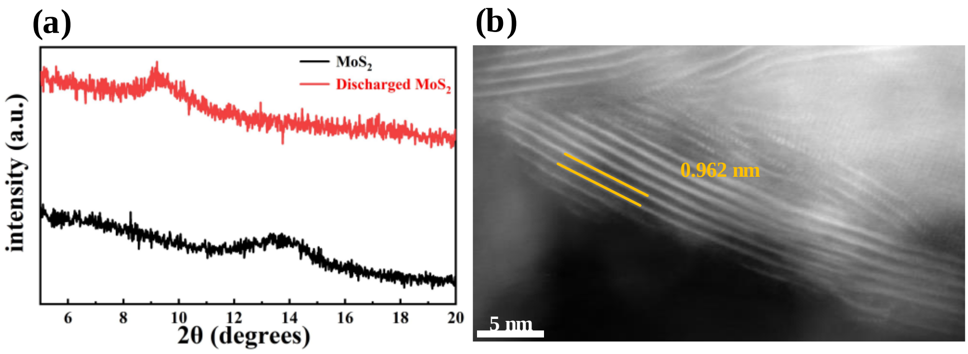

Fig. S28 The **a**) XRD pattern and **b**) HTEM image of MoS_2 cathodes after fully discharged

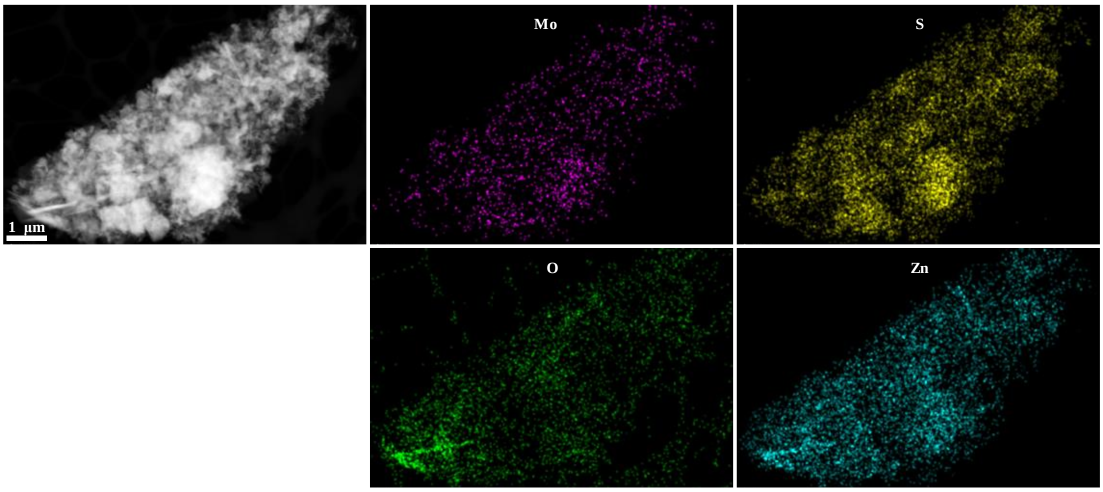

Fig. S29 STEM elemental mapping image of MoS_2 cathodes after fully discharged

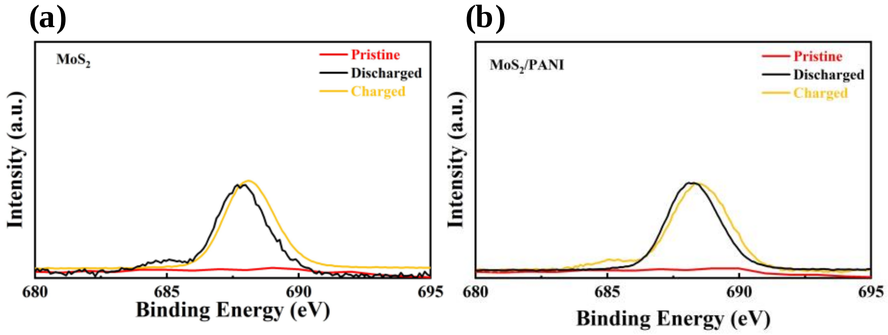

Fig. S30 F 1s XPS spectrum of pristine, fully discharged and charged states of **a**) original MoS_2 and **b**) MoS_2/PANI cathode

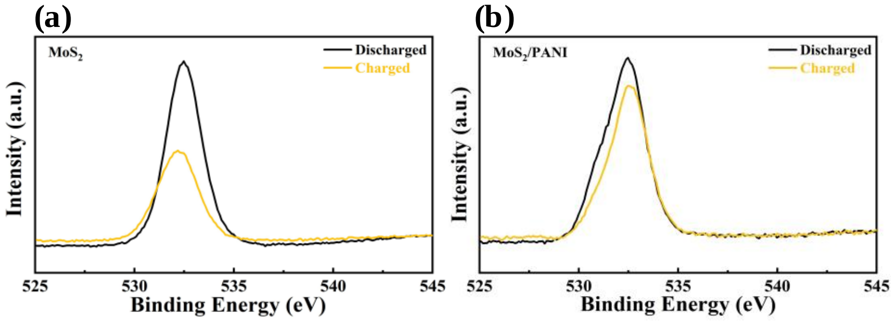

Fig. S31 O 1s XPS spectrum of pristine, fully discharged and charged states of **a**) original MoS_2 and **b**) MoS_2/PANI cathode

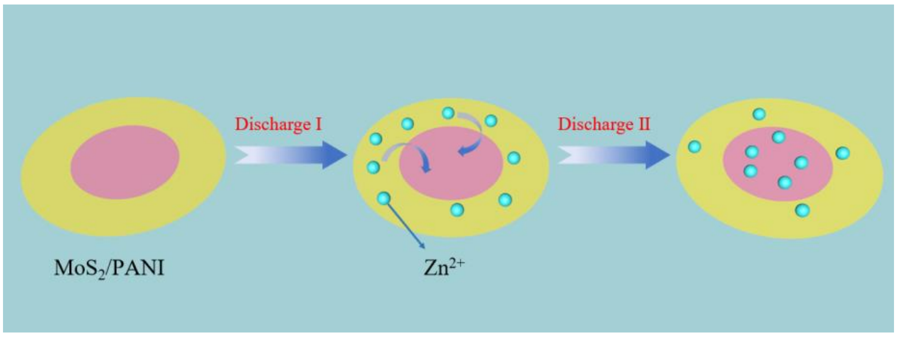

Fig. S32 Zn^{2+} stored procedure in MoS_2/PANI cathode

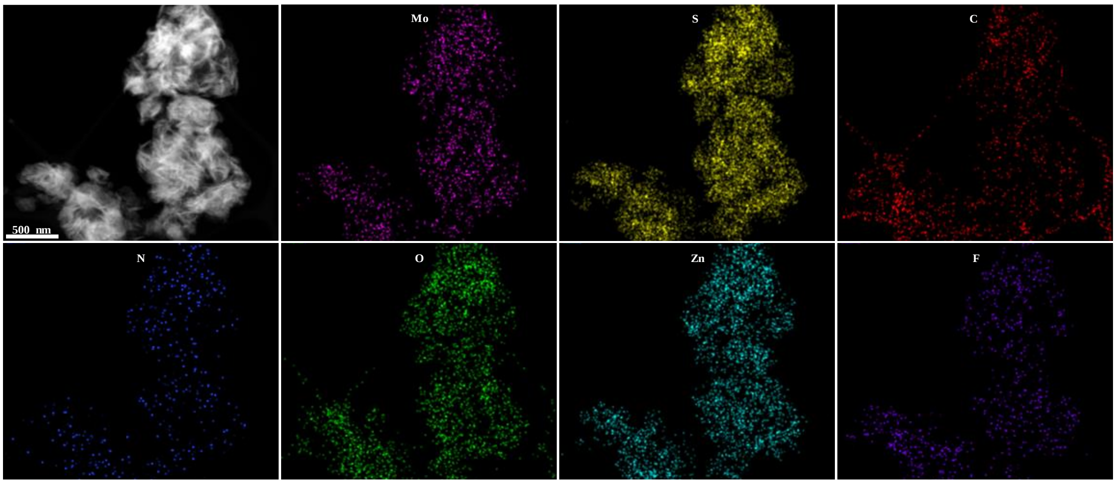

Fig. S33 The STEM elemental mapping image of $Zn_{0.85-x}$Mo/P after immersed in 2 M $Zn(CF_3SO_3)_2$ for 24.0 h with Air

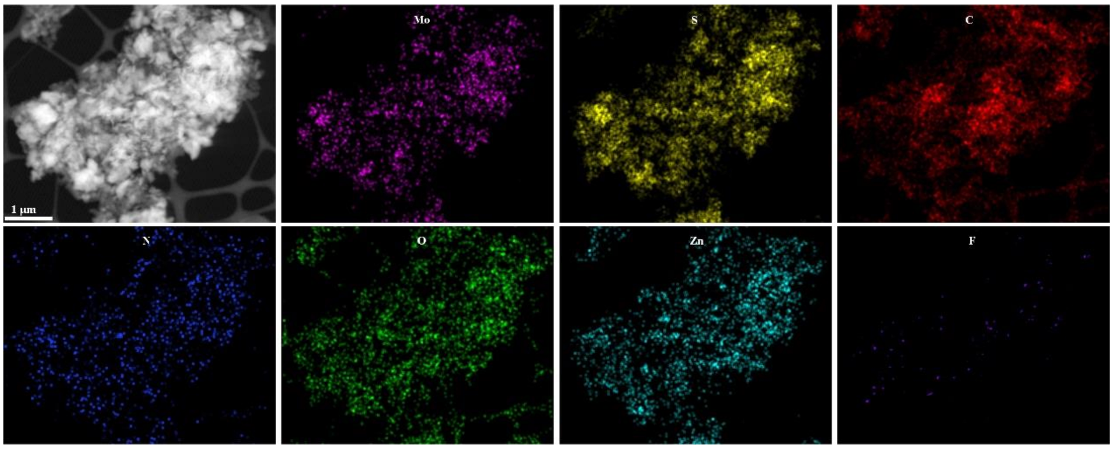

Fig. S34 STEM elemental mapping image of $Zn_{0.85-x}$Mo/P after immersed in 2 M $Zn(CF_3SO_3)_2$ for 24.0 h with N_2

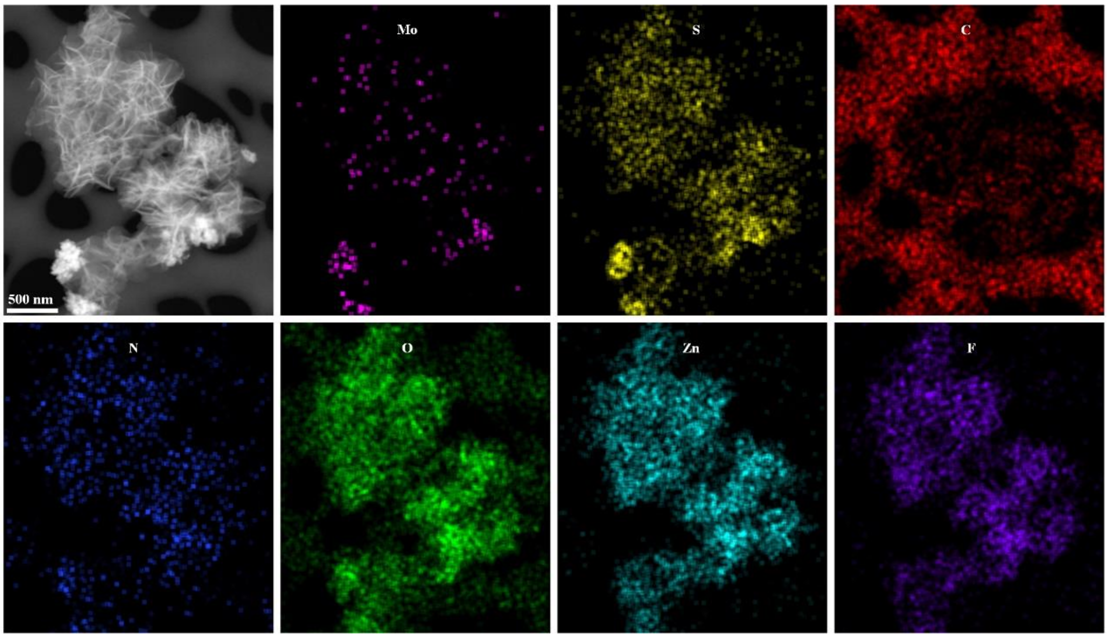

Fig. S35 STEM elemental mapping image of $Zn_{x+y}(CF_3SO_3)_{2y}(OH)_{2x}$ (with a little MoS_2)

The content of F element on the surface of $Zn_{0.85-x}Mo/P$ electrode in the presence of air is much higher than that in the presence of N_2, which is attributed to the formation of $Zn_{x+y}(CF_3SO_3)_{2y}(OH)_{2x}$. In addition, the $Zn_{x+y}(CF_3SO_3)_{2y}(OH)_{2x}$ was observed in the presence of air (Fig. S34). These evidences prove that O_2 and H_2O combine to form OH^- when $Zn_{0.85-x}Mo/P$ is air recharging. Then the generated OH- and extracted Zn^{2+} combine with the adsorbed electrolyte ions (Zn^{2+} and $CF_3SO_3^{2-}$) to form an amorphous trifluoride containing the layered dihydroxide $Zn_{x+y}(CF_3SO_3)_{2y}(OH)_{2x}$.

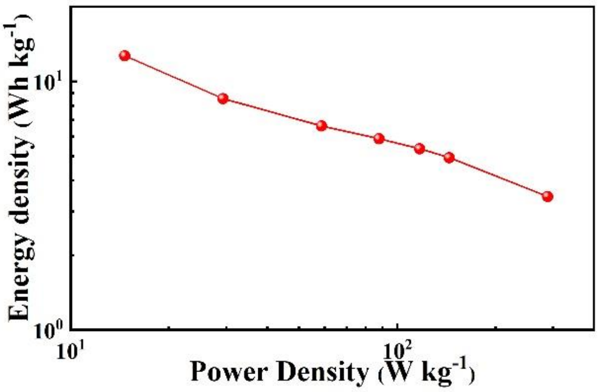

Fig. S36 The energy and power density plot based the mass of the two electrodes

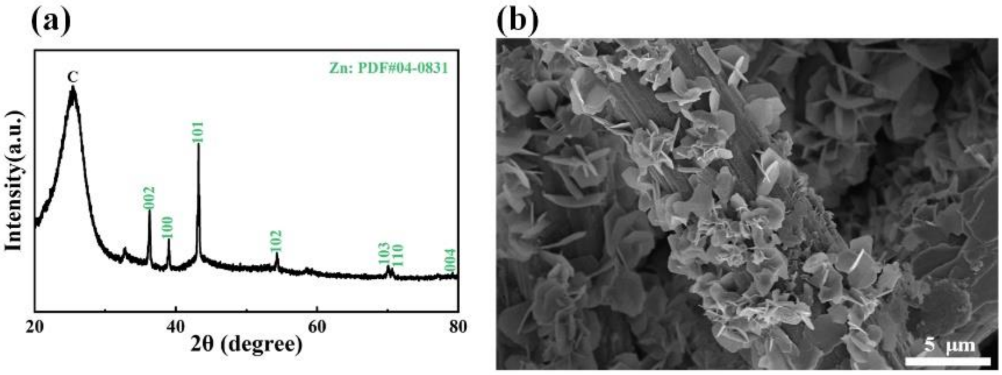

Fig. S37 a) XRD pattern and **b**) SEM image of zinc nanoflakes

Table S1 XPS fitting results of Mo 3d in original MoS_2 and MoS_2/PANI cathodes peaks at different reaction potentials

Sample Status	Mo^{6+} (%)	2H (%)	1T (%)
original MoS_2	5.00	29.18	65.82
MoS_2/PANI	4.39	22.87	72.74
Charged	6.59	23.86	69.55
Discharged	15.48	18.78	65.74
4.0 h	10.60	20.73	68.67
24.0 h	8.81	21.92	69.27

The embedding of a small amount of PANI is the main reason for the phase transition.

Table S2 *R*s and *R*ct of MoS_2, PANI and MoS_2/PANI fitting from the EIS spectra

Materials	*R*s (Ω)	*R*ct (Ω)
MoS_2	10.241	41.442
PANI	9.922	15.312
MoS_2/PANI	9.938	28.112

Table S3 Comparison of the Zn^{2+} diffusion coefficient of MoS_2/PANI cathodes with manganese and vanadium-based oxides in the aqueous systems

Cathode Material	D_{Zn}	References
Layered MnO_2	10^{-12}-10^{-14} cm^2 s^{-1}	[S9]
Al-ion modified $V_2O_5{\cdot}1.6H_2O$	10^{-11}-10^{-13} cm^2 s^{-1}	[S10]
V_2O_5	10^{-9}-10^{-11} cm^2 s^{-1}	[S11]
MnO_2/MnO@C	~10^{-10}-10^{-11} cm^2 s^{-1}	[S12]
MoS_2	10^{-11}-10^{-12} cm^2 s^{-1}	**This Work**
MoS_2/PANI	**10^{-9} to 10^{-11} cm^2 s^{-1}**	**This Work**

Table S4 ICP analyses of discharged and charged MoS_2/PANI cathode

Material	chemical element	Concentration (mg/L)	Molar ratio (Zn/Mo)
discharged MoS_2/PANI	Mo	6.543	0.8474
	Zn	3.754	
charged MoS_2/PANI	Mo	6.053	0.0237
	Zn	0.097	

Supplementary References

[S1] S. Li, Y. Liu, X. Zhao, K. Cui, Q. Shen et al., Molecular engineering on mos2 enables large interlayers and unlocked basal planes for high-performance aqueous zn-ion storage. Angew Chem. Int. Ed. **60**(37), 20286-20293 (2021). https://doi.org/10.1002/anie.202108317

[S2] H. Liang, Z. Cao, F. Ming, W. Zhang, D.H. Anjum et al., Aqueous zinc-ion storage in MoS_2 by tuning the intercalation energy. Nano Lett. **19**(5), 3199-3206 (2019). https://doi.org/10.1021/acs.nanolett.9b00697

[S3] J. Liu, P. Xu, J. Liang, H. Liu, W. Peng et al., Boosting aqueous zinc-ion storage in MoS_2 via controllable phase. Chem. Eng. J. **389**, 124405 (2020). https://doi.org/10.1016/j.cej.2020.124405

[S4] H. Liu, J.-G. Wang, W. Hua, Z. You, Z. Hou et al., Boosting zinc-ion intercalation in hydrated MoS_2 nanosheets toward substantially improved performance. Energy Storage Mater. **35** 731-738 (2021). https://doi.org/10.1016/j.ensm.2020.12.010

[S5] W. Xu, C. Sun, K. Zhao, X. Cheng, S. Rawal et al., Defect engineering activating (boosting) zinc storage capacity of MoS_2. Energy Storage Mater. **16,** 527-534 (2019). https://doi.org/10.1016/j.ensm.2018.09.009

[S6] H. Li, Q. Yang, F. Mo, G. Liang, Z. Liu et al., MoS_2 nanosheets with expanded interlayer spacing for rechargeable aqueous zn-ion batteries. Energy Storage Mater. **19,** 94-101 (2019). https://doi.org/10.1016/j.ensm.2018.10.005

[S7] S. Li, Y. Liu, X. Zhao, Q. Shen, W. Zhao et al., Sandwich-like heterostructures of MoS_2/graphene with enlarged interlayer spacing and enhanced hydrophilicity as high-performance cathodes for aqueous zinc-ion batteries. Adv. Mater. **33**(12), e2007480 (2021). https://doi.org/10.1002/adma.202007480

[S8] C. Li, C. Liu, Y. Wang, Y. Lu, L. Zhu et al., Drastically-enlarged interlayer-spacing MoS_2 nanocages by inserted carbon motifs as high performance cathodes for aqueous zinc-ion batteries. Energy Storage Mater. **49** 144-152 (2022). https://doi.org/10.1016/j.ensm.2022.03.048

[S9] J. Huang, Z. Wang, M. Hou, X. Dong, Y. Liu et al., Polyaniline-intercalated

manganese dioxide nanolayers as a high-performance cathode material for an aqueous zinc-ion battery. Nat Commun. **9**, (2018). https://doi.org/10.1038/s41467-018-04949-4

[S10] J. Zheng, C. Liu, M. Tian, X. Jia, E.P. Jahrman et al., Fast and reversible zinc ion intercalation in al-ion modified hydrated vanadate. Nano Energy **70**((2020). https://doi.org/10.1016/j.nanoen.2020.104519

[S11] X. Chen, L. Wang, H. Li, F. Cheng, J. Chen, Porous V_2O_5 nanofibers as cathode materials for rechargeable aqueous zinc-ion batteries. J. Energy Chem. **38**, 20-25 (2019). https://doi.org/10.1016/j.jechem.2018.12.023

[S12] W. Jiang, H. Shi, X. Xu, J. Shen, Z. Xu et al., Mno stabilized in carbon-veiled multivariate manganese oxides as high-performance cathode material for aqueous Zn-ion batteries. Energy Environ. Mater. **4**(4), 603-610 (2020). https://doi.org/10.1002/eem2.12142
